# Bioactive natural products predominantly derived from Chinese herbal medicines for diminished ovarian reserve: mechanistic insights into ovarian protection

**DOI:** 10.3389/fendo.2026.1942723

**Published:** 2026-09-09

**Authors:** Ze Ma, Lan Wang, Li Tang, Jiacheng Zhang, Ruifan Lin, Xichen Chai, Yutian Zhu, Yang Ye, Xiyan Xin, Dong Li

**Affiliations:** State Key Laboratory of Female Fertility Promotion, Department of Traditional Chinese Medicine, Peking University Third Hospital, Beijing, China

**Keywords:** bioactive monomers, diminished ovarian reserve, ovarian protection, oxidative stress, traditional Chinese medicine

## Abstract

Diminished ovarian reserve (DOR) is characterized by a reduction in oocyte quantity and quality and involves complex, interconnected pathological mechanisms. This review systematically evaluates bioactive natural products, predominantly derived from Chinese herbal medicines, using a mechanism-first framework, while considering their chemical scaffolds and botanical sources as complementary descriptors. Current evidence indicates that these compounds exert ovarian-protective effects through coordinated regulation of oxidative stress, inflammatory responses, DNA-damage repair and cellular senescence, apoptosis-related signaling, ferroptosis, and autophagy-mitophagy homeostasis. Across granulosa-cell systems and animal models of chemotherapy-induced ovarian injury, irradiation, accelerated aging, and reproductive aging, representative compounds have demonstrated beneficial effects on endocrine function, follicular preservation, mitochondrial homeostasis, oocyte quality, and fertility-related outcomes. Their capacity to modulate multiple interacting pathways may be particularly valuable for DOR, given its multifactorial pathogenesis. Emerging findings further suggest that the effects of apoptosis-, autophagy-, and growth-related signaling are dependent on cell type, pathway status, dose, timing, and disease stage, highlighting restoration of ovarian homeostasis as a central therapeutic principle. Although further studies incorporating rigorous causal validation, ovarian pharmacokinetic characterization, and standardized experimental design are warranted, the available evidence supports these natural products as promising candidates for mechanistic investigation and future therapeutic development in DOR.

## 1 Introduction

DOR refers to a decline in both the number of available oocytes and their developmental competence, ultimately resulting in impaired reproductive potential and endocrine dysfunction (1, 2). Clinically, DOR is commonly characterized by decreased anti-Müllerian hormone (AMH) levels, elevated follicle-stimulating hormone (FSH) levels, reduced antral follicle count (AFC), and poor ovarian responsiveness during assisted reproduction. In recent years, the burden of DOR has continued to increase, particularly among women of advanced reproductive age and those exposed to genetic, autoimmune, environmental, surgical, or iatrogenic factors, including chemotherapy and radiotherapy. Epidemiological evidence further suggests that the prevalence of DOR rises markedly with age, highlighting its increasing significance in reproductive medicine and women’s health (3).

The pathogenesis of DOR is complex and multifactorial. Current evidence indicates that follicular depletion, dysregulated hormonal homeostasis, oxidative stress, mitochondrial dysfunction, apoptosis, impaired autophagy, inflammatory responses, and granulosa cell injury all contribute to the progressive decline in ovarian function. Among these mechanisms, oxidative stress is considered a major driver of ovarian aging. Excessive production of reactive oxygen species (ROS) disrupts intracellular redox homeostasis, damages mitochondrial structure and oxidative phosphorylation, reduces ATP generation, and promotes granulosa cell injury and follicular atresia. Meanwhile, age-related impairment of autophagic flux and reduced resistance to oxidative stress may further aggravate DNA damage, cellular dysfunction, and abnormal follicular development, thereby accelerating the loss of ovarian reserve. In addition, mitochondrial dysfunction has increasingly been recognized as a key determinant of ovarian aging, as abnormalities in mitochondrial quantity, morphology, and bioenergetic capacity are closely associated with reduced oocyte quality and compromised fertility (4, 5).

Current therapeutic strategies for DOR mainly include hormone replacement therapy, ovulation induction, assisted reproductive technologies, stem cell-based interventions, and other supportive treatments. Although these approaches may temporarily improve hormonal profiles or reproductive outcomes, they are unable to fundamentally reverse ovarian aging. Therefore, there remains an urgent need to develop safer and more effective interventions targeting the molecular basis of ovarian dysfunction.

**Figure 1:**
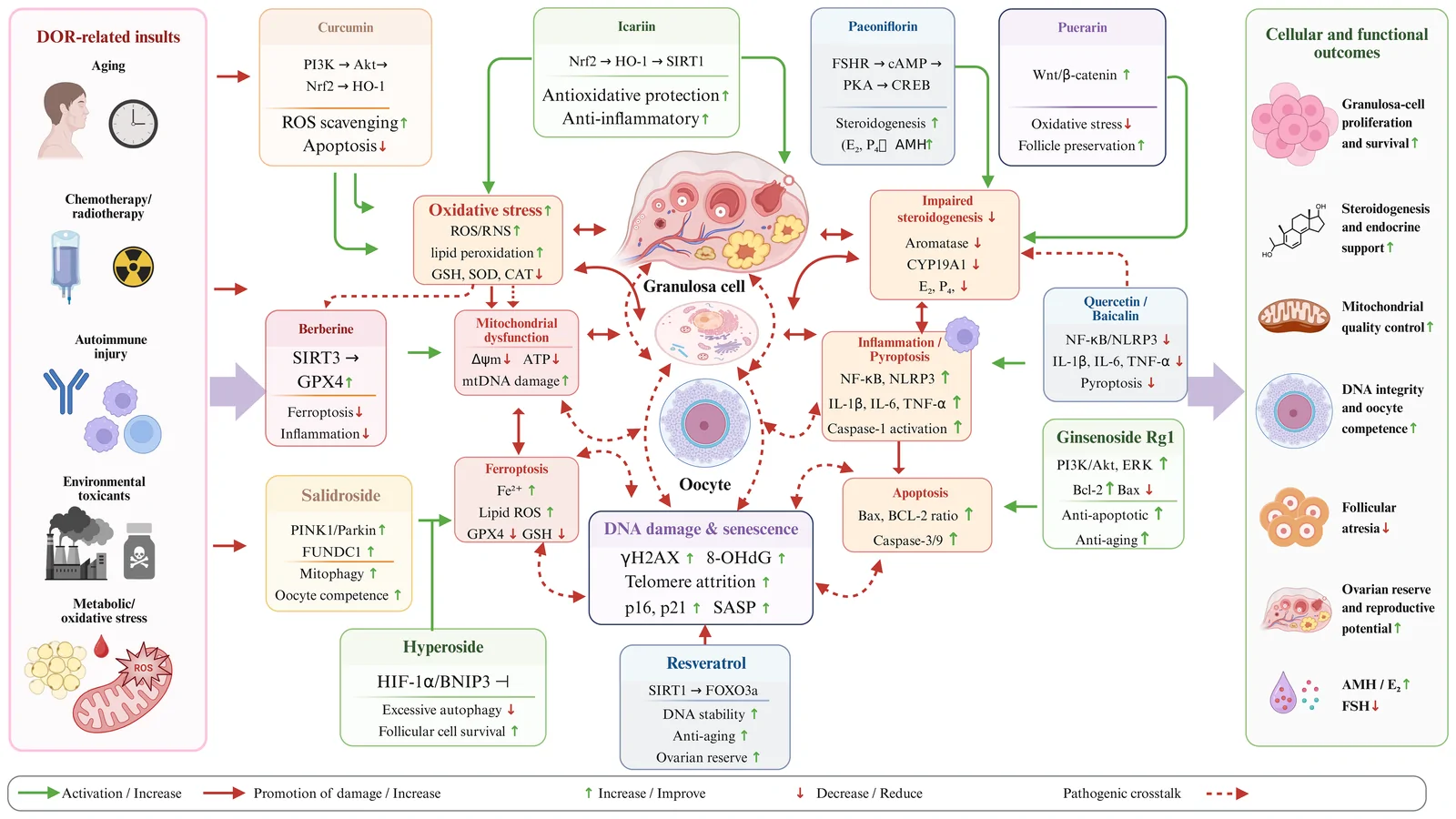
Mechanistic overview of ovarian functional decline and the protective actions of bioactive natural products and related small molecules.

Traditional Chinese Medicine (TCM) has attracted increasing attention in ovarian dysfunction because of its multitarget pharmacology, and purified bioactive compounds derived from Chinese medicinal herbs are particularly amenable to standardization and mechanistic investigation. DOR, premature ovarian insufficiency (POI), and poor ovarian response (POR) should nevertheless be distinguished. DOR denotes reduced follicle quantity and/or oocyte competence; POI denotes loss of ovarian activity before 40 years of age; and POR describes a suboptimal response to controlled ovarian stimulation. DOR, POI, and POR represent sequential or interrelated facets of ovarian dysfunction rather than subordinate categories, with the present review centered on DOR. Because direct DOR intervention studies remain limited, POI, POR-relevant ovarian dysfunction, chemotherapy- or irradiation-induced ovarian injury, and ovarian-aging models are used as lateral, mechanistically supportive evidence rather than as subsets of DOR. The review evaluates whether natural products predominantly derived from Chinese herbal medicines preserve ovarian function through redox control, inflammation suppression, DNA-damage repair, apoptosis regulation, ferroptosis control, and autophagy/mitophagy homeostasis, while explicitly considering model relevance, cell type, dose, timing, and evidentiary limitations (**Figure 1**).

### 1.1 Etiology and pathological mechanisms of DOR

DOR is characterized by an early decline in both the quantity and quality of ovarian follicles before the expected age-related depletion of the ovarian pool, leading to reduced fecundity, impaired ovarian responsiveness, and endocrine disturbance. Its etiology is multifactorial and involves not only physiological ovarian aging, but also a broad spectrum of pathological contributors, including genetic and chromosomal susceptibility, autoimmune injury, environmental toxicant exposure, and iatrogenic damage induced by chemotherapy, radiotherapy, pelvic surgery, or other gonadotoxic interventions. Age remains the dominant determinant of ovarian reserve decline, as the primordial follicle pool is finite and continuously lost through follicular activation and atresia; however, accelerated depletion may occur when intrinsic repair capacity is weakened or when the ovary is exposed to inflammatory, oxidative, or toxic insults (5–8).

At the mechanistic level, excessive apoptosis and dysfunction of granulosa cells are central events in DOR progression, because granulosa cells are essential for follicular survival, steroidogenesis, oocyte metabolic support, and maintenance of the follicular microenvironment. Once granulosa-cell homeostasis is disrupted, follicular atresia accelerates and ovarian reserve declines (7, 9). Oxidative stress is one of the most consistently implicated upstream drivers: excessive ROS impair mitochondrial bioenergetics, damage mtDNA and nuclear DNA, disturb spindle integrity and meiosis, and activate apoptosis-related pathways, thereby compromising both oocyte competence and follicular survival (5, 7). In parallel, dysregulation of the PI3K/AKT/mTOR–FOXO axis may promote aberrant primordial follicle activation or impair follicular quiescence, ultimately leading to premature exhaustion of the follicular pool (7).

Chronic low-grade inflammation also appears to be an important contributor to ovarian aging and DOR. Aging ovaries exhibit a pro-inflammatory microenvironment characterized by increased macrophage and lymphocyte infiltration, upregulation of inflammatory cytokines such as IL-1β, IL-6, and TNF-α, and activation of inflammasome-related pathways, all of which may aggravate follicular injury and accelerate follicle loss (8). In addition, autoimmune oophoritis and related endocrine autoimmunity can damage steroidogenic cells and growing follicles, further diminishing ovarian reserve in susceptible women (6). Environmental pollutants, including persistent organic pollutants and perfluoroalkyl substances, may also impair ovarian reserve by inducing oxidative stress, mitochondrial dysfunction, epigenetic disturbance, and steroidogenic defects (5).

Furthermore, emerging evidence suggests that impaired ovarian angiogenesis and stromal microenvironment remodeling are also involved in DOR pathogenesis. Reduced vascular support limits oxygen and nutrient delivery to follicles, whereas extracellular matrix disorganization and stromal fibrosis may mechanically and biochemically restrict follicle growth and ovulation (8). These pathological processes are accompanied clinically by hormonal alterations, most commonly elevated FSH and reduced AMH, reflecting both follicular depletion and disturbed hypothalamic–pituitary–ovarian axis regulation (9). Collectively, these interconnected pathways—follicular overactivation, granulosa-cell apoptosis, oxidative damage, mitochondrial dysfunction, chronic inflammation, immune dysregulation, angiogenic impairment, and stromal remodeling—jointly drive the progressive decline in ovarian reserve and reproductive potential in women with DOR.

## 2 Literature search strategy

**Figure 2:**
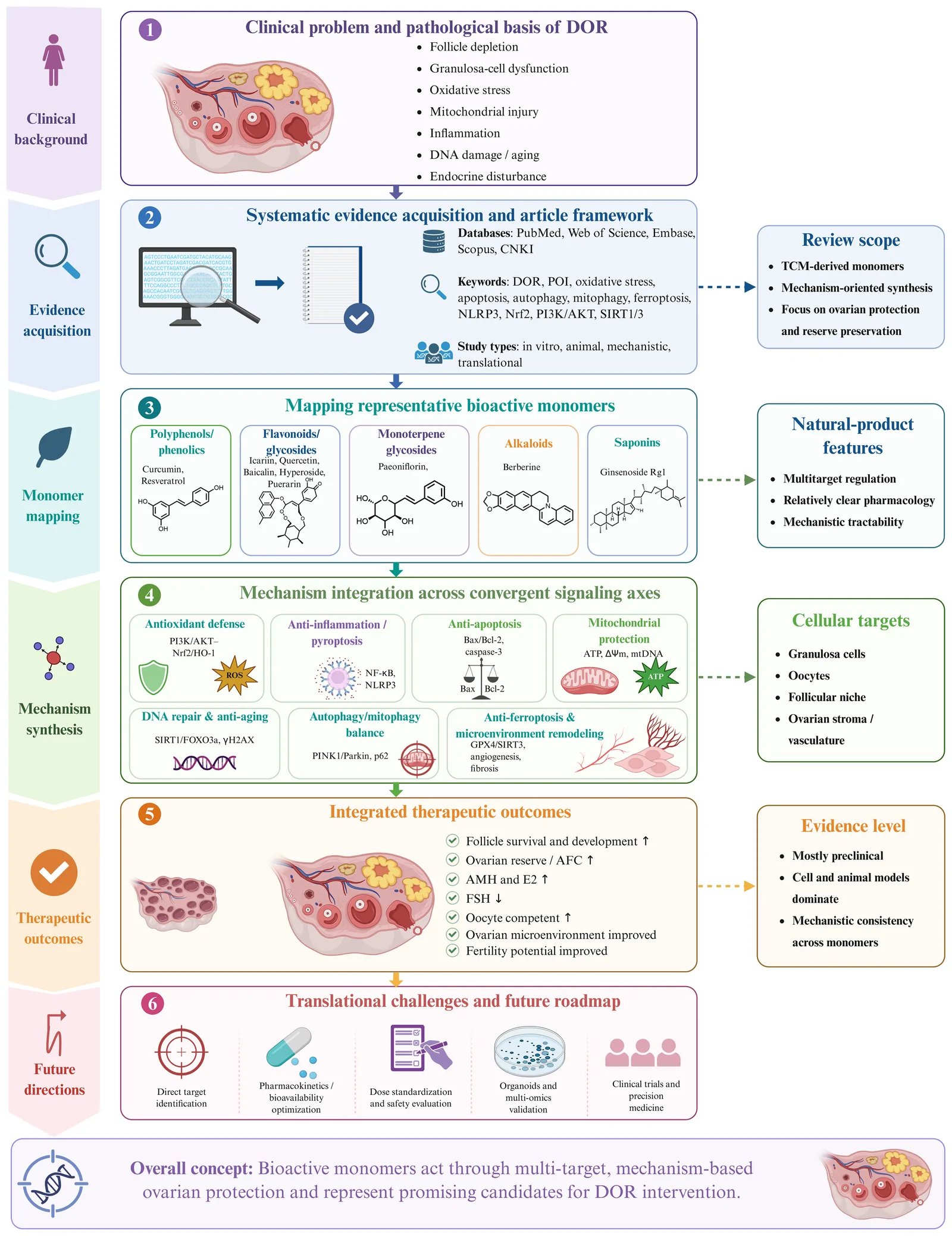
Roadmap of the review framework integrating evidence acquisition, monomer classification, mechanistic synthesis, and translational perspectives.

This review was developed through a structured literature search combined with reference tracking and citation expansion. Databases included PubMed, Web of Science Core Collection, Embase, Scopus, the Cochrane Library, China National Knowledge Infrastructure (CNKI), Wanfang Data, and the Chinese Science and Technology Journal Database (VIP). Controlled vocabulary and free-text terms covered diminished ovarian reserve (DOR), ovarian aging, premature ovarian insufficiency (POI), poor ovarian response (POR), Traditional Chinese Medicine (TCM), phytochemical, monomer, flavonoid, polyphenol, saponin, iridoid, oxidative stress, apoptosis, autophagy, mitophagy, ferroptosis, pyroptosis, inflammation, Nrf2, PI3K/Akt, mTOR, SIRT1, SIRT3, AMPK, FOXO3a, NLRP3, and representative compounds. The narrative synthesis was organized primarily by functional mechanism; chemical scaffold and botanical source were applied as secondary descriptors (**Figure 2**).

The inclusion criteria were as follows:

1. studies directly related to DOR, ovarian aging, chemotherapy-induced ovarian injury, or follicular depletion;
2. studies clearly elucidating molecular mechanisms or signaling pathways;
3. priority given to studies published within the past five years;
4. articles with retrievable DOI numbers.

Structured appraisal of preclinical evidence and methodological considerations.

To improve the transparency and interpretability of the heterogeneous preclinical literature, we conducted a structured, compound-specific appraisal of model relevance and methodological robustness. As summarized in **Table 1**, we extracted the species and cell type, model type and underlying injury mechanism, targeted pathways, target-validation strategies, dose range, route of administration, treatment duration, and available dose–response information for each compound, together with major limitations related to sample-size reporting, control design, causal target validation, and pharmacokinetic or ovarian-exposure data. DOR-specific models were distinguished from chemotherapy-, irradiation-, accelerated-aging-, reproductive-aging-, POI-, and POR-related models because these systems reproduce different aspects of ovarian dysfunction and are not directly interchangeable. In interpreting the findings, greater weight was placed on studies reporting concordant cellular and *in vivo* effects, multiple doses or dose–response relationships, direct target engagement, loss-of-function or rescue experiments, appropriate controls, and clearly described experimental procedures. Studies limited to phenotypic improvement or changes in pathway-associated markers were interpreted more cautiously. Accordingly, the available findings are discussed in a model-, cell-type-, dose-, timing-, and pathway-state-dependent manner and should be regarded as preclinical mechanistic support rather than evidence of established clinical efficacy.

**Table 1:** Compound-specific preclinical evidence-support appraisal in DOR-related experimental systems.

Compound	Model / injury / cell type	Dose, route, duration / dose-response	Target-validation method
Paeoniflorin (10, 11)	Cisplatin DOR mice; KGN cells; chemotherapy injury	(10) In vivo: 10 mg/kg/day, i.p., 7 weeks (single dose) (11). In vivo: 75 and 150 mg/kg/day, intragastric gavage, 4 weeks; in vitro: 1, 10, 20, and 50 μM, 48 h (multi-dose; dose-dependent).	FSHR knockdown/antagonism plus pathway and phenotypic assays
Puerarin (12)	Cyclophosphamide + busulfan mouse POF model	(12) In vivo: 100 and 200 mg/kg/day, gavage, 28 days (multi-dose; dose-related efficacy).	Wnt/β-catenin, Nrf2/SOD2 and Bcl-2/Bax marker concordance; no rescue
Curcumin (13, 19)	D-galactose mouse and cyclophosphamide rat ovarian-failure models	(13) In vivo: 100 mg/kg/day, i.p., 42 days (19). In vivo: 100 mg/kg/day for 14 days; route NR (single dose in each study; no within-study dose-response).	Nrf2/HO-1 and PI3K/Akt marker changes; no genetic/pharmacologic rescue
Ginsenoside Rg1 (14)	D-galactose POI mice; accelerated-aging injury	(14) In vivo: 20 mg/kg/day, i.p., 28 days (single dose).	p21/p53/STK and Bax/Bcl-2 expression; no target-engagement test
Berberine (15, 33, 55)	Chemotherapy-injury animals; autoimmune POI and DHT-injured human GC evidence	(15) In vivo: 50 mg/kg; route, frequency, and duration NR in accessible report (33). In vitro: 50 μM, 24 h (single concentration) (55). In vitro: 4, 8, and 12 μM, 24 h (multi-concentration).	Inflammatory/redox/SIRT3 pathway assays; no direct ligand-target validation
Icariin (16, 32, 38)	Cisplatin mice/KGN cells; autoimmune or D-galactose POI models	(16) In vivo: 30 mg/kg/day, i.p., 21 days; in vitro: 5 μg/mL, 6 h (32). In vivo: 40 mg/kg/day, 28 days; route NR (38). In vivo: 10, 50, and 100 mg/kg/day, i.p., 42 days; in vitro: 100 nM, 1 μM, and 10 μM, 6 h (multi-dose; dose-dependent).	Nrf2 loss/inhibition and Keap1 docking in one study; DDR/pathway assays in others
Hyperoside (17)	Cyclophosphamide mice and KGN cells	(17) In vivo: 40 mg/kg/day, intragastric gavage, 5 weeks; in vitro: 10 μM, duration NR (single dose/concentration).	HIF-1α/BNIP3 autophagy assays; no causal rescue/direct engagement
Isoquercitrin (20)	AAPH-injured human ovarian granulosa cells; oxidative injury	(20) In vitro: 100 μM, 24 h (single concentration).	Antioxidant, HO-1 and mitochondrial phenotyping; no loss-of-function/rescue
Genistein (21)	Ionizing-radiation ovarian-injury animal model	(21) In vivo: 5 mg/kg/day, i.p., 10 days (single dose).	ER-β/TGF-β/FOXL2 and apoptotic markers; no direct engagement
Baicalin (30)	H_2_O_2_-injured KGN granulosa-like cells	(30) In vitro: 0–80 μM screen, 24 h; 20 μM used for mechanistic assays (non-monotonic; benefit attenuated above 40 μM and 80 μM was inhibitory).	USP48-related expression and inflammatory/oxidative phenotypes; no rescue
Eriodictyol (31)	Mouse premature ovarian-failure model	(31) In vivo: 20, 40, and 80 mg/kg/day, oral gavage, 28 days (multi-dose; strongest effect at 80 mg/kg).	PI3K/Akt/NF-κB markers and macrophage/inflammatory outcomes
Quercetin (34, 82)	Cyclophosphamide mice and primary granulosa cells	(34) No original experimental regimen (secondary review) (82). In vivo: 12.5, 25, and 50 mg/kg/day, intragastric gavage, 4 weeks (multi-dose; dose-related improvement).	Mitochondrial and pyroptosis assays; no direct ligand-target/rescue study
Resveratrol (39, 40)	POF animals; cisplatin model used in combination with melatonin	(39) In vivo: 20 and 40 mg/kg/day, gavage, 28 days (multi-dose; dose-dependent) (40). Combination-study dose, route, duration, and comparable dose-response NR in accessible report.	SIRT1/FOXO3a/BCL2 and DNA-damage markers; no direct engagement
Pterostilbene (54)	H_2_O_2_-injured human ovarian granulosa cells	(54) Tested cellular concentration(s) and duration NR in accessible report (cell only).	Nrf2/HO-1, ferroptosis and mitochondrial assays; no rescue/direct binding
Salidroside (64)	Reproductively aged mice and oocytes	(64) In vivo efficacy: 50 mg/kg, i.p., 14-day course (frequency NR); PK: single 50 mg/kg i.p. dose (single efficacy dose).	Transcriptomics, microproteomics and mitophagy assays
Spermidine (22, 59)	Oxidative-injury and reproductive-aging mice; high-dose toxicity evidence	(22) In vivo: 10 and 15 mg/kg; route and duration NR (multi-dose) (59). In vivo: 25, 50, and 100 mg/kg i.p. dose screen; 50 mg/kg/day i.p. used in main experiments; duration NR (non-monotonic; 50 mg/kg outperformed 25 and 100 mg/kg) (88). In vivo: 150 mg/kg i.p., acute single exposure; assessed at 24 h (harmful).	Nrf2/HO-1/GPX4, Akt/FHC/ACSL4 and mitophagy assays; positive and negative dose evidence
NMN (58)	Age-related DOR mice; granulosa-cell mitophagy	(58) In vivo: 0.5 mg/mL in drinking water for 20 weeks (40–60 weeks of age; single concentration).	Mitophagy and mitochondrial-homeostasis assays; no direct engagement
Melatonin (40, 67)	Cisplatin combination model and BPS ovarian-toxicity model	(40) Combined-treatment dose, route, and duration NR in accessible report (67). Dose, route, and duration NR in accessible report; no comparable dose-response extractable.	SIRT1/FOXO3a and autophagy/mitophagy markers; no direct target test
NAC (48)	D-galactose-injured rabbit ovarian granulosa cells	(48) In vitro: 0.5 mg/mL for 12 h, selected as the optimal concentration after screening; higher concentrations showed reduced benefit (exact tested series NR; non-monotonic).	PI3K/Akt/mTOR and mitochondrial-apoptosis assays; no rescue/direct engagement
IU1 (41)	Human biochemical-POI granulosa cells plus D-galactose POI mice	(41) In vitro: 50 μM pretreatment for 24 h. In vivo: 40 mg/kg/day i.p. for 14 days before ETO, and 40 mg/kg/day i.p. for 6 weeks in the D-gal model (single dose; two durations).	Defined USP14 inhibition with DDR/NHEJ readouts and in vivo confirmation
EGCG (83)	Cyclophosphamide mouse ovarian-tissue injury	(83) In vivo: 5, 25, and 50 mg/kg/day i.p. for 3 days before CTX (multi-dose; 25 and 50 mg/kg effective).	p-Akt/FOXO3a/rpS6 and inflammatory/apoptotic assays; no rescue
Honokiol (84)	Radiation-induced POF mice	(84) In vivo: 10 mg/kg/day i.p. for 7 days (selected regimen; complete screened-dose series NR).	Nrf2/HO-1 and apoptosis/pyroptosis assays; no causal rescue
Daphnetin (85)	D-galactose wild-type and Nrf2-knockout mice	(85) In vivo: 15 and 30 mg/kg/day i.p. for 42 days (multi-dose; dose-dependent).	Nrf2 genetic loss-of-function with ovarian phenotyping
Chrysin (86)	D-galactose mouse ovarian-failure model	(86) In vivo: 36.1, 72.2, and 144.4 mg/kg/day, co-administered during the 42-day D-gal modeling period; route NR (multi-dose; dose-dependent).	Redox, inflammatory and apoptotic markers; no causal rescue/direct engagement

## 3 Natural product origins of bioactive monomers used in DOR research

**Figure 3:**
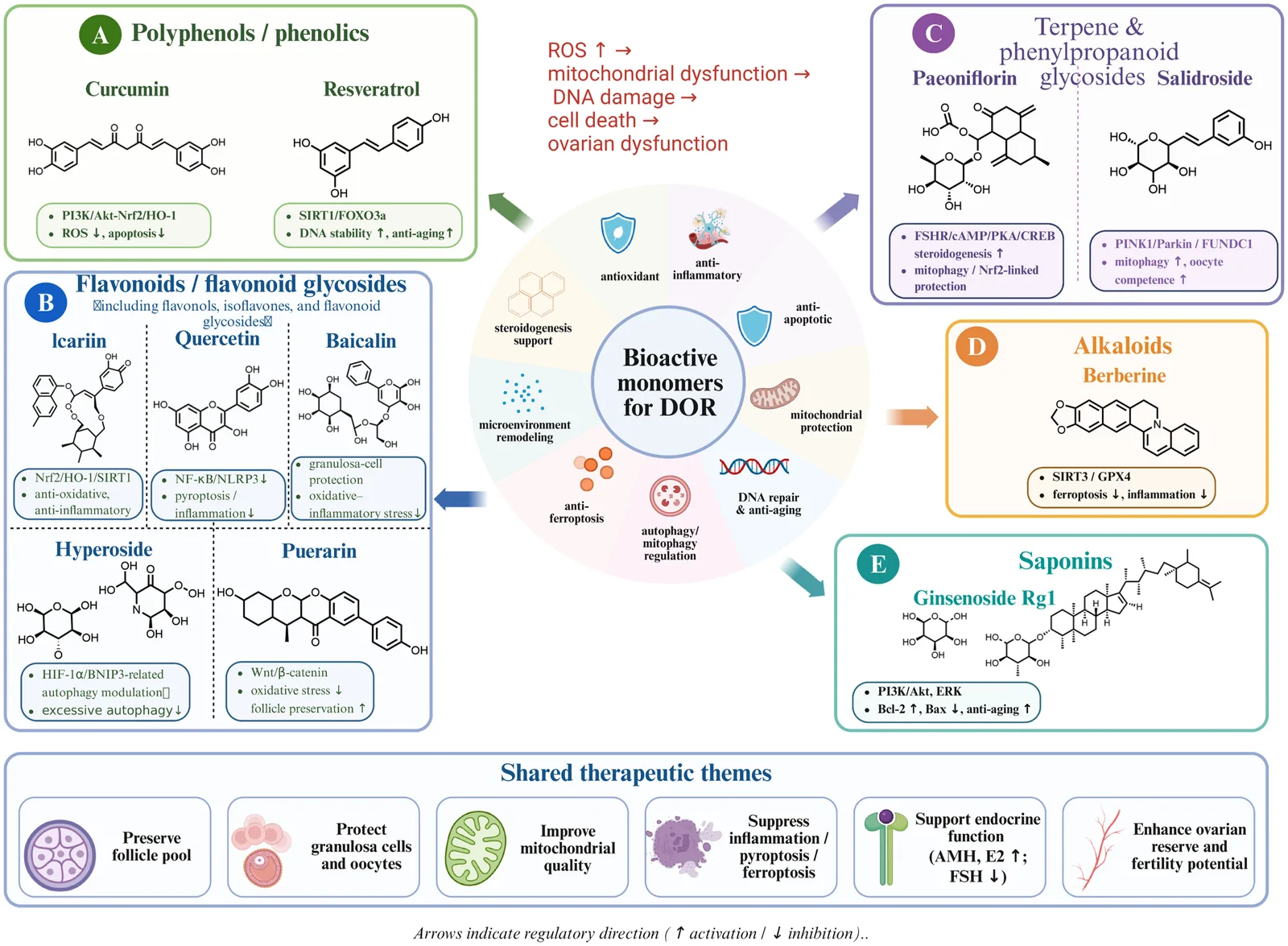
Representative bioactive monomers grouped by chemical class and their major ovarian-protective mechanisms. **(A)** Polyphenols/phenolics. **(B)** Flavonoids/flavonoid glycosides. **(C)** Terpene and phenylpropanoid glycosides. **(D)** Alkaloids. **(E)** Saponins.

The review uses a two-level classification. Functional mechanism is the primary organizing principle for the narrative sections, whereas chemical class and botanical source are secondary descriptors used in **Table 2** and **Figure 3**. A compound may appear in more than one mechanistic domain when separate experiments support pleiotropic activity, but it is not assigned simultaneously to unrelated top-level taxonomies. This hierarchy avoids mixing chemical categories with functional pathways.

**Table 2:** Major bioactive monomers and related small molecules involved in ovarian protection.

Drug / bioactive compound	Main source / category	Main mechanisms / pathways	Main ovarian protective effects
Paeoniflorin	Paeonia lactiflora; monoterpene glycoside	FSHR/cAMP/PKA/CREB; Nrf2/HO-1; mitophagy	Improves granulosa-cell function, restores hormone levels, and reduces oxidative injury and apoptosis.
Puerarin	Pueraria lobata; isoflavone	Wnt/beta-catenin; Nrf2/SOD2; Bcl-2/Bax	Increases follicle number, attenuates oxidative stress, and suppresses apoptosis.
Curcumin	Curcuma longa; diarylheptanoid/polyphenol	Nrf2/HO-1; PI3K/Akt; antioxidant and anti-ferroptotic signaling	Improves ovarian reserve-related indices and alleviates oxidative damage and granulosa-cell apoptosis.
Ginsenoside Rg1	Panax ginseng; triterpenoid saponin	Antioxidant and anti-inflammatory regulation; p21/p53-related senescence signaling	Ameliorates ovarian pathological injury and delays ovarian aging-related changes.
Berberine	Coptis and Berberis species; isoquinoline alkaloid	NF-kappaB inhibition; Nrf2 activation; SIRT3-related signaling	Improves ovarian reserve-related indices and reduces inflammation and oxidative stress.
Icariin	Epimedium species; flavonoid glycoside	Nrf2/ARE; Nrf2/HO-1/Sirt1; DNA damage repair	Suppresses ferroptosis and apoptosis and improves ovarian function.
Hyperoside	Flavonol glycoside from multiple medicinal plants	HIF-1alpha/BNIP3-mediated autophagy regulation	Reduces follicular depletion and improves fertility-related outcomes.
Isoquercitrin	Flavonoid glycoside; active component of Rubi fructus	HO-1-associated antioxidant defense; mitochondrial protection	Enhances granulosa-cell viability and reduces oxidative injury.
Genistein	Soy-derived isoflavone	ER-beta/TGF-beta-related signaling; mitochondrial apoptotic pathway	Protects follicles and helps restore AMH and estradiol levels.
Baicalin	Scutellaria baicalensis; flavonoid glycoside	USP48-related inflammatory-oxidative stress regulation	Promotes granulosa-cell proliferation and reduces inflammation and apoptosis.
Eriodictyol	Flavonoid	PI3K/Akt/NF-kappaB	Improves endocrine and histological injury in ovarian dysfunction models.
Quercetin	Plant-derived flavonoid	NF-kappaB; NLRP3; caspase-1/GSDMD-related pyroptosis signaling	Attenuates inflammation, oxidative stress, and pyroptosis-related ovarian injury.
Resveratrol	Stilbene polyphenol from grapes, Polygonum cuspidatum, and other sources	SIRT1/FOXO3a; antioxidant and anti-inflammatory regulation	Increases follicle number and alleviates DNA damage-related ovarian injury.
Pterostilbene	Stilbene derivative related to resveratrol	Nrf2/HO-1; anti-ferroptotic regulation	Protects granulosa cells and reduces lipid peroxidation.
Salidroside	Rhodiola-derived phenylethanoid glycoside	Mitophagy regulation	Improves oocyte quality in reproductive aging models.
Spermidine	Natural polyamine	Nrf2/HO-1/GPX4; Akt/FHC/ACSL4; mitophagy	Reduces ferroptosis and improves oocyte quality and developmental competence.
Nicotinamide mononucleotide (NMN)	NAD+ precursor	Mitophagy enhancement	Improves age-related decline in ovarian reserve by supporting mitochondrial homeostasis.
Melatonin	Endogenous indoleamine	SIRT1/FOXO3a; autophagy/mitophagy regulation	Reduces follicular atresia and protects against DNA damage.
N-acetylcysteine (NAC)	Antioxidant small molecule; glutathione precursor	PI3K/Akt/mTOR; mitochondrial apoptosis inhibition	Improves granulosa-cell survival and suppresses mitochondrial apoptosis.
USP14 inhibitor (IU1)	Targeted small-molecule inhibitor	DNA damage response; NHEJ -related repair signaling	Promotes DNA repair and delays granulosa -cell senescence.
Epigallocatechin-3-gallate (EGCG)	Camellia sinensis; catechin	Akt/FOXO3a/rpS6-associated signaling; anti-inflammatory and anti-apoptotic effects	Attenuates chemotherapy-related follicular damage, inflammation, and apoptosis.
Honokiol	Magnolia officinalis; biphenolic natural product	Nrf2/HO-1-associated antioxidant signaling; apoptosis and pyroptosis regulation	Alleviates radiation-associated ovarian injury and supports ovarian function.
Daphnetin	Daphne species; coumarin	Nrf2-dependent antioxidant and antisenescence signaling	Improves follicular and endocrine outcomes in an accelerated-aging model.
Chrysin	Natural flavone	Antioxidant, anti-inflammatory, and anti-apoptotic regulation	Reduces ovarian injury and improves functional indices in an accelerated-aging model.

NMN, melatonin, NAC, and IU1 are related small molecules or mechanistic auxiliary evidence rather than classical TCM-derived monomers. Rows added in response to Reviewer 2, Comment 10 are shown in red.

Preliminary structure–activity considerations remain hypothesis-generating. Phenolic hydroxylation and catechol-like motifs in flavonoids and polyphenols may favor reactive-oxygen-species scavenging and metal chelation; glycosylation in hyperoside, isoquercitrin, and puerarin can alter solubility, permeability, and metabolism; prenylation in icariin increases lipophilicity and may affect membrane or protein interactions; conjugated polyphenolic scaffolds in curcumin and stilbenoids can influence antioxidant and electrophile-responsive signaling; and saponin or alkaloid scaffolds, such as ginsenoside Rg1 and berberine, have distinct membrane and protein-interaction profiles. These relationships should not be considered established ovarian SARs because comparative analog series, direct binding measurements, and ovarian tissue pharmacokinetics are generally unavailable.

The compound scope was broadened with additional representative natural products associated with Chinese medicinal sources. Quercetin produced dose-dependent protection in cyclophosphamide-induced ovarian insufficiency and was linked to mitochondrial biogenesis and suppression of granulosa-cell pyroptosis (10). Epigallocatechin-3-gallate (EGCG), a Camellia sinensis catechin, reduced cyclophosphamide-associated ovarian inflammation and apoptosis with changes in Akt/FOXO3a/rpS6 signaling (11). Honokiol from Magnolia officinalis improved radiation-induced ovarian injury through Nrf2/HO-1-associated antioxidant and antipyroptotic effects (12). Daphnetin, a coumarin associated with Daphne species, showed Nrf2-dependent protection in a D-galactose model, including genetic loss-of-function evidence (13). Chrysin likewise attenuated oxidative, inflammatory, and apoptotic injury in D-galactose-induced ovarian failure (14). These additions broaden chemical coverage without removing the recurrent limitations of model specificity, incomplete target validation, and absent ovarian exposure data.

From the perspective of pharmacognosy and natural product chemistry, the bioactive monomers currently investigated for DOR and related ovarian insufficiency states are not confined to a single medicinal category but are instead distributed across several recurrent botanical source lineages enriched in ovarian-protective phytochemicals (**Table 2**). One major source lineage comprises root-and rhizome-derived medicinal plants, which provide a large proportion of the best-studied candidate compounds, including paeoniflorin from *Paeonia lactiflora* (15, 16), puerarin from Pueraria lobata (17), curcumin from Curcuma longa (18), ginsenoside Rg1 from Panax ginseng (19)and berberine from Coptis and related Berberis species (20). A second source lineage is represented by kidney-tonifying aerial herbs, among which icariin, a characteristic prenylated flavonoid from Epimedium species, is one of the most extensively studied examples in chemotherapy-related ovarian injury models (21). A third source lineage includes polyphenol-and flavonoid-rich medicinal or food–medicine homologous botanical resources, represented by hyperoside, a flavonol glycoside widely distributed in medicinal plants such as *Crataegus pinnatifida*, *Forsythia suspensa*, and *Cuscuta chinensis* (22). Collectively, these monomers span multiple major natural-product classes, including monoterpene glycosides, isoflavones, diarylheptanoids, triterpenoid saponins, isoquinoline alkaloids, and flavonoid glycosides, indicating that candidate monomers for DOR intervention are highly diverse in both botanical origin and structural scaffold.

Despite their heterogeneous plant origins and chemical backbones, these monomers converge on a relatively consistent pharmacological profile in ovarian protection, most commonly involving anti-oxidative, anti-apoptotic, anti-inflammatory, mitochondrial-protective, and granulosa cell-supportive activities. Representative evidence supports this shared framework. Paeoniflorin has been shown to alleviate cisplatin-induced DOR by restoring granulosa-cell steroidogenic function through activation of the FSHR/cAMP/PKA/CREB pathway (16), while also promoting ovarian development under oxidative injury by activating mitophagy and enhancing Nrf2/HO-1-associated antioxidant defense (15). Puerarin improves follicular morphology and ovarian function in experimental ovarian insufficiency, with reported involvement of Wnt/β-catenin-related regulation and oxidative stress attenuation (17). Curcumin exerts protective effects against ovarian failure by suppressing oxidative stress and granulosa-cell apoptosis through Nrf2/HO-1 and PI3K/Akt signaling (18). Ginsenoside Rg1 ameliorates pathological ovarian injury in aging-related POI models by enhancing anti-oxidant capacity, reducing inflammatory cytokines, and modulating senescence-associated pathways (19). Berberine similarly improves ovarian reserve and endocrine disturbance in chemotherapy-induced POI, at least in part through coordinated suppression of oxidative stress and inflammatory signaling (20). Icariin protects against cisplatin-induced ovarian damage by inhibiting oxidative stress, ferroptosis, and apoptosis via activation of the Nrf2/ARE pathway (21). Hyperoside has also demonstrated ovarian-protective activity by attenuating cyclophosphamide-induced follicular depletion and fertility impairment through suppression of HIF-1α/BNIP3-mediated excessive autophagy (22). Taken together, current evidence indicates that the principal natural reservoirs for DOR-related monomer discovery are traditional medicinal roots and rhizomes, kidney-tonifying herbs, and flavonoid-rich botanical resources, whereas their dominant mechanistic themes consistently center on redox homeostasis, mitochondrial quality control, anti-apoptotic signaling, preservation of granulosa-cell function, and maintenance of follicular homeostasis (**Figure 3**).

### 3.1 Regulation of oxidative stress and the Nrf2 signaling pathway

Oxidative stress is widely recognized as one of the core pathogenic events driving DOR, because excessive accumulation of reactive oxygen species (ROS) disrupts the delicate redox balance required for follicular survival, steroidogenesis, and oocyte competence. Under physiological conditions, moderate ROS participate in ovulation, follicular angiogenesis, and intracellular signaling; however, once ROS generation exceeds endogenous antioxidant capacity, oxidative damage to lipids, proteins, mitochondrial DNA, and nuclear DNA accumulates, ultimately leading to mitochondrial dysfunction, loss of mitochondrial membrane potential, impaired ATP production, granulosa-cell apoptosis, and accelerated follicular atresia (5).

In ovarian aging and POI/DOR-related injury, the Keap1/Nrf2/ARE axis serves as a central endogenous defense system against oxidative damage. During oxidative challenge, Nrf2 dissociates from Keap1, translocates into the nucleus, and activates antioxidant response element (ARE)-driven genes, including HO-1, NQO1, SOD, CAT, GPX, and other glutathione-related enzymes, thereby restoring redox homeostasis and limiting oxidative injury in granulosa cells and oocytes (15). Impairment of this pathway has been associated with weakened antioxidant defense, enhanced inflammatory amplification, and progressive depletion of ovarian reserve, indicating that Nrf2-centered antioxidant signaling represents an important mechanistic target in monomer-based intervention for DOR (5, 23).

Accumulating evidence indicates that several natural monomers can ameliorate ovarian oxidative injury by directly or indirectly reinforcing Nrf2-related antioxidant signaling. Curcumin is one of the most representative examples. In a D-galactose-induced premature ovarian failure model, curcumin increased ovarian SOD activity, reduced MDA accumulation, lowered 8-OHdG, 4-HNE, and NTY expression, alleviated granulosa-cell apoptosis, and upregulated p-Akt, Nrf2, and HO-1, suggesting coordinated activation of the PI3K/Akt and Nrf2/HO-1 pathways (18). In a cyclophosphamide-induced model, curcumin likewise improved ovarian oxidative stress markers, restored AMH and estradiol levels, reduced FSH/LH elevation, and ameliorated histopathological damage, further supporting its antioxidant and ovarian-protective potential (24). Isoquercitrin, identified as the most effective Rubi fructus component in a 2,2′-azobis(2-amidinopropane) dihydrochloride (AAPH)-injured human granulosa-cell model, reduced intracellular ROS, improved mitochondrial membrane potential, decreased apoptosis, enhanced glutathione peroxidase-related antioxidant activity, and ameliorated HO-1-associated oxidative defense, indicating a marked ability to preserve granulosa-cell viability under oxidative challenge (25). Hyperoside also showed significant protective effects in chemotherapy-related ovarian damage: although its dominant mechanism was described as suppression of HIF-1α/BNIP3-mediated excessive autophagy, it simultaneously restored mitochondrial membrane potential, reduced apoptosis, improved estrous cyclicity and ovarian reserve indices, and therefore attenuated oxidative stress-associated follicular injury (22). Genistein, a soy-derived monomer, preserved primordial and growing follicles after irradiation, restored AMH and estradiol, elevated endogenous glutathione and glutathione peroxidase activity, and suppressed Bax/cytochrome c/caspase-3-mediated mitochondrial apoptosis, demonstrating that reinforcement of antioxidant defense can effectively interrupt ROS-driven follicular loss (26). More recently, spermidine has been shown to alleviate ovarian oxidative damage by activating the Nrf2/HO-1/GPX4 pathway while concurrently modulating Akt/FHC/ACSL4 signaling, thereby reducing granulosa-cell apoptosis, attenuating ferroptosis, improving endocrine function, increasing AMH-defined ovarian reserve, and preserving reproductive capacity (27). In addition, deer blood peptides were reported to increase SOD content, decrease MDA accumulation, upregulate Nrf2 and HO-1 while suppressing Keap1, and reverse the imbalance of Bcl-2/Bax/caspase-3 signaling in a D-gal-induced premature ovarian failure model, further supporting the concept that antioxidant and anti-apoptotic modulation of the Nrf2/HO-1 axis can preserve follicular structure and ovarian function under oxidative stress (28). Taken together, these monomers and bioactive compounds converge on a common protective theme: they reduce ROS overload, maintain mitochondrial integrity, enhance antioxidant enzyme systems, and suppress oxidative stress-induced granulosa-cell death through Nrf2-centered or Nrf2-associated signaling, thereby delaying follicular depletion and preserving ovarian reserve in DOR-related conditions.

### 3.2 Anti-inflammatory effects and regulation of the inflammasome

Chronic low-grade inflammation is increasingly recognized as a major driver of ovarian aging and the progression of DOR. Persistent inflammatory activation in the aging ovary is accompanied by immune-cell infiltration, stromal remodeling, cellular senescence, and fibrosis, which together disrupt follicular homeostasis and accelerate the decline in oocyte quality and ovarian responsiveness (29–32). Among the inflammatory pathways implicated in this process, the NLRP3 inflammasome has emerged as a central mechanistic node linking metabolic stress, oxidative damage, and follicular depletion. Activation of NLRP3 promotes caspase-1 cleavage and subsequent maturation of IL-1β and IL-18, thereby amplifying NF-κB-dependent inflammatory signaling and driving inflammatory programmed cell death, including pyroptosis (33). In addition, local metabolic overload may further aggravate this process at the granulosa-cell level. Elevated glucose in the follicular microenvironment has been shown to activate NLRP3-dependent pyroptosis in ovarian granulosa cells, accompanied by increased caspase-1 activation, IL-1β release, and GSDMD-associated membrane injury, suggesting that the inflammasome–pyroptosis axis directly contributes to a hostile ovarian inflammatory microenvironment (34). Collectively, these findings indicate that chronic inflammation in DOR is not merely a secondary phenomenon, but rather an active pathological process that interacts with oxidative stress, mitochondrial dysfunction, and fibro-inflammatory remodeling to accelerate follicular atresia and ovarian insufficiency.

Within this pathological framework, natural monomers derived from traditional Chinese medicine often exhibit a multi-target anti-inflammatory pattern by simultaneously suppressing cytokine cascades, attenuating oxidative stress, and correcting the ovarian immune microenvironment. Baicalin has been shown to reduce IL-1β, IL-6, IL-8, and TNF-α levels while enhancing granulosa-cell proliferation and limiting apoptosis in H2O2-injured KGN cells, at least partly through regulation of USP48-related inflammatory–oxidative stress coupling (35). Eriodictyol also exerts clear ovarian protection in chemotherapy-induced ovarian dysfunction by suppressing the PI3K/Akt/NF-κB pathway, reducing macrophage-mediated granulosa-cell injury, and improving endocrine and histological indices (36). In premature ovarian insufficiency models, berberine attenuates ovarian damage by inhibiting NF-κB signaling while enhancing Nrf2-associated antioxidant defense, thereby reducing inflammatory injury and improving ovarian structure and hormone levels (20). Icariin displays additional immunomodulatory activity in autoimmune-related ovarian insufficiency, where it upregulates Nrf2/HO-1/Sirt1 signaling, promotes Treg-associated immune tolerance, and mitigates immune-mediated follicular damage (37). Beyond direct DOR/POI models, berberine has also been shown to suppress inflammation in ovarian disease through inhibition of hyaluronan synthase 2 and related inflammatory signaling, providing further support for its anti-inflammatory pharmacological potential (38). In addition, polyphenolic flavonoids such as quercetin have been increasingly recognized for their capacity to modulate inflammatory, oxidative, and pyroptosis-related pathways in ovarian dysfunction, including downregulation of NF-κB, IL-1β, IL-6, TNF-α, NLRP3, caspase-1, and GSDMD-related signaling, thereby suggesting a broader role for flavonoid monomers in interrupting the “inflammation–pyroptosis–follicular depletion” cascade (39). Taken together, these bioactive monomers converge on a common therapeutic principle: they alleviate chronic ovarian inflammation by suppressing inflammasome-related signaling, restraining NF-κB-centered cytokine amplification, reducing immune-cell-mediated granulosa-cell injury, and improving the inflammatory microenvironment, thereby helping preserve follicular survival and ovarian reserve in DOR-related conditions.

### 3.3 DNA damage repair and anti-aging signaling regulation

Among the emerging mechanisms underlying monomer-based intervention in DOR, preservation of genomic stability and modulation of aging-related signaling networks have become increasingly important. Current evidence suggests that ovarian reserve decline is not merely a quantitative loss of follicles, but a complex pathological process involving persistent DNA damage, impaired DNA damage response (DDR), oxidative stress, telomere attrition, mitochondrial dysfunction, and premature senescence of oocytes and granulosa cells (40–42). Because oocytes remain arrested for prolonged periods and granulosa cells are continuously exposed to metabolic and inflammatory stress, both cell types are highly vulnerable to cumulative genotoxic injury. Once DNA lesions exceed repair capacity, checkpoint activation, apoptosis, and senescence are induced, ultimately accelerating follicular atresia and ovarian functional decline (40–42).

Within this framework, icariin provides one of the clearest examples of a monomeric compound directly linked to ovarian DNA repair. In a D-galactose-induced premature ovarian failure model, icariin improved ovarian morphology, increased follicle numbers, restored endocrine indices including estradiol and AMH, and enhanced fertility outcomes. More importantly, icariin reduced the expression of γH2AX and 53BP1 in granulosa cells, indicating that its ovarian protective effect was closely associated with attenuation of DNA double-strand break injury and promotion of DNA damage repair (43). These findings are highly relevant to DOR, in which defective repair of cumulative DNA lesions is increasingly regarded as a central mechanism driving reserve exhaustion (40, 43).

Curcumin also fits well within the DNA protection–anti-aging framework. In a D-galactose-induced ovarian failure model, curcumin improved ovarian function, preserved the primordial follicle pool, reduced granulosa-cell apoptosis, and attenuated oxidative injury. Mechanistically, curcumin decreased 8-OHdG and p16 expression and activated the Nrf2/HO-1 and PI3K/Akt pathways, suggesting that it not only alleviates oxidative DNA injury but also suppresses senescence-associated signaling and reinforces pro-survival responses (18). Thus, curcumin may help preserve ovarian reserve by reducing the upstream burden of oxidative genomic damage and interrupting its downstream conversion into apoptosis and aging phenotypes (18).

Resveratrol represents another important monomer acting along this axis. Experimental evidence indicates that resveratrol improves ovarian morphology, increases follicle number, reduces follicular atresia, relieves oxidative stress and inflammation, and promotes female germline stem-cell survival in premature ovarian failure models (44). Although the main study emphasis was placed on antioxidative and anti-inflammatory protection, these effects are mechanistically relevant to genomic stability because chronic oxidative and inflammatory stress are major upstream drivers of DNA lesions and persistent DDR activation in the aging ovary. Accordingly, resveratrol can be reasonably interpreted as a monomer capable of indirectly preserving ovarian reserve by stabilizing the cellular environment in which DNA integrity is maintained (44).

Ginsenoside Rg1 likewise demonstrates how anti-aging signaling intersects with preservation of ovarian function. In D-galactose-induced premature ovarian insufficiency models, Rg1 improved ovarian and uterine morphology, restored ovarian weight, enhanced antioxidant enzyme activities, reduced inflammatory cytokines, and significantly decreased the expression of senescence-related factors including p21 and p53 (19). Since persistent DDR signaling commonly converges on p53/p21-mediated cell-cycle arrest and senescence, these data suggest that Rg1 may alleviate ovarian aging by restraining the downstream execution of DNA damage-associated senescence programs (19).

Further support for this concept comes from combined melatonin and resveratrol treatment. In a cisplatin-induced premature ovarian failure model, co-administration of melatonin and resveratrol more effectively reduced follicular atresia and pH2AX-positive DNA damage signals than either agent alone, while simultaneously increasing SIRT1, FOXO3a, and BCL2 expression (45). These results indicate that modulation of the SIRT1/FOXO3a/BCL2 axis may help coordinate DNA damage attenuation, anti-apoptotic signaling, and stress resistance within the ovary. Although this study involved combination therapy rather than a single monomer alone, it strongly supports the view that targeting the DNA damage–aging signaling network is an effective strategy for preserving ovarian reserve (45).

More direct evidence that DDR itself is therapeutically actionable comes from recent work on USP14 inhibition. In granulosa cells from biochemical POI patients, USP14 was found to be upregulated, while inhibition with IU1 decreased etoposide-induced DNA damage, promoted DDR, enhanced non-homologous end joining, increased RNF168, Ku70, DDB1 and 53BP1-related repair responses, and suppressed granulosa-cell senescence (46). *In vivo*, IU1 also ameliorated the D-galactose-induced POI phenotype. Although IU1 is a targeted small-molecule inhibitor rather than a classical phytochemical monomer, this study is highly informative for DOR mechanism review because it demonstrates in a causal manner that improving granulosa-cell DNA repair competence can directly delay ovarian functional decline (46).

At the broader mechanistic level, mitochondrial impairment should also be considered part of the DNA damage–aging network. A review focused on ovarian mitochondrial aging emphasizes that age-related mitochondrial dysfunction reduces ATP supply, weakens ROS-scavenging capacity, promotes oxidative stress, and facilitates apoptosis and follicular depletion, while nucleus–mitochondria crosstalk may represent a therapeutic target in ovarian aging (47). This notion helps explain why monomeric compounds with antioxidant, mitochondrial-protective, or sirtuin-modulating properties may exert effects beyond simple ROS reduction, extending instead to preservation of genomic stability and ovarian cellular homeostasis (41, 45, 47).

In addition, evidence from ovarian aging models further supports a causal link between accumulated DNA damage and reserve decline. Growth hormone-related ovarian aging research has shown that increased γH2AX-associated DNA double-strand breaks in oocytes and granulosa cells are accompanied by greater macrophage infiltration and adverse effects on the ovarian reserve, whereas lower DNA damage is associated with a more preserved reserve phenotype (48). Although this is not a monomer intervention study, it provides valuable auxiliary support for the central proposition that suppressing DNA damage accumulation and senescence signaling is biologically meaningful for maintaining ovarian reserve (48).

Collectively, available evidence suggests that monomer-based therapy may delay ovarian reserve decline through a multilayered mechanism centered on genomic maintenance and anti-aging signaling regulation. At the upstream level, these agents reduce oxidative and inflammatory stress that initiates DNA lesions; at the intermediate level, they suppress markers such as γH2AX, 53BP1, 8-OHdG, or pH2AX and may enhance core DDR machinery; and at the downstream level, they inhibit senescence-and apoptosis-related pathways such as p53/p21 while reinforcing pro-survival networks including SIRT1/FOXO3a/BCL2, PI3K/Akt, and Nrf2/HO-1. Therefore, maintenance of DNA integrity, enhancement of repair competence, and reprogramming of ovarian aging signaling should be regarded as an important theoretical foundation for the development of monomer interventions in DOR.

### 3.4 Regulation of apoptosis and the PI3K/Akt/mTOR signaling pathway

Granulosa cell apoptosis is widely regarded as a critical event in the progression of DOR, because granulosa cells provide indispensable metabolic support, steroidogenic signals, and survival cues for oocyte maintenance and follicular maturation. Once granulosa-cell homeostasis is disrupted, mitochondrial dysfunction, oxidative stress, and activation of intrinsic apoptotic machinery progressively impair follicular integrity, ultimately leading to follicular atresia and accelerated depletion of ovarian reserve (16, 49). At the molecular level, this process is closely associated with mitochondrial outer membrane permeabilization, increased Bax/Bcl-2 imbalance, cytochrome c release, and activation of the downstream Caspase cascade, whereas the PI3K/Akt/mTOR pathway serves as a central regulatory hub coordinating granulosa-cell proliferation, survival, autophagy, and follicle activation (18, 49). Dysregulation of this pathway may therefore contribute to DOR through two interrelated routes: by enhancing granulosa-cell apoptosis and by disturbing the balance between follicular dormancy and activation, thereby accelerating follicular exhaustion (15, 49).

Among the uploaded studies, paeoniflorin provides particularly relevant evidence for DOR-oriented intervention. In cisplatin-induced DOR mice and granulosa-like KGN cells, paeoniflorin improved estrous cyclicity, ovarian index, serum estradiol levels, antral follicle and corpus luteum numbers, and restored granulosa-cell steroidogenic capacity through activation of the FSHR/cAMP/PKA/CREB pathway (16). Although that study focused mainly on endocrine recovery, a second recent study demonstrated that paeoniflorin directly suppresses ovarian cell apoptosis under oxidative stress conditions, decreases Bax expression, increases Bcl-2 expression, reduces TUNEL-positive granulosa cells, and attenuates γH2A-associated DNA damage while promoting granulosa-cell proliferation (15). Taken together, these findings suggest that paeoniflorin exerts a dual protective role in ovarian insufficiency: it enhances granulosa-cell functional responsiveness to gonadotropins while simultaneously limiting oxidative stress-associated apoptotic injury, thereby preserving follicular development and ovarian reserve (15, 16).

Puerarin likewise exhibits anti-apoptotic and follicle-preserving properties, although its principal mechanistic focus in the uploaded study was the Wnt/β-catenin pathway rather than PI3K/Akt directly. In cyclophosphamide-and busulfan-induced premature ovarian failure, puerarin increased total follicle number and primordial follicle ratio, reduced follicular atresia, restored Oct4 and Mvh expression, enhanced antioxidant defenses including SOD2 and Nrf2, and increased the Bcl-2/Bax ratio (17). These results indicate that puerarin suppresses intrinsic apoptotic signaling and improves follicular survival under gonadotoxic stress. Although this study does not directly establish PI3K/Akt/mTOR activation, the improvement in mitochondrial apoptotic balance and oxidative stress status strongly supports the broader concept that monomeric compounds can preserve ovarian reserve by restraining granulosa-cell apoptosis and stabilizing pro-survival intracellular signaling networks (17).

Metformin is another related small molecule relevant to this section, particularly because it links ovarian reserve protection to PI3K/Akt/mTOR-mediated survival signaling. In a cyclophosphamide-induced ovarian reserve injury model in prepubertal mice, metformin protected ovarian reserve through regulation of the PI3K/Akt/mTOR signaling pathway and Yap-1 (50). Although metformin is not a traditional herbal monomer, this evidence is mechanistically relevant because PI3K/Akt/mTOR signaling is closely involved in granulosa-cell survival, apoptosis regulation, and follicular maintenance (50).

More direct pathway-level evidence is provided by a recent N-acetylcysteine (NAC) study in ovarian granulosa cells. In D-galactose-induced granulosa-cell injury, NAC suppressed apoptosis, decreased Bax, p53, and caspase-9 expression, increased BCL-2 expression, reduced mitochondrial cytochrome c release, improved CAT, GSH, and SOD activity, and modulated the PI3K/Akt/mTOR pathway (51). Although NAC is not a traditional herbal monomer, this cell-based work provides mechanistic support for interpreting antioxidant regulation of survival signaling; it does not establish efficacy, pharmacokinetics, or target engagement in an intact DOR ovary.

At the disease-mechanism level, evidence from human DOR granulosa cells further confirms that PI3K/Akt/mTOR signaling is tightly linked to granulosa-cell proliferation and apoptosis. A recent study showed that lncRNA-THBS4 expression in granulosa cells correlates positively with AMH, AFC, and good embryo rate and negatively with basal FSH in women with DOR; mechanistically, silencing lncRNA-THBS4 inhibited PI3K, Akt, and mTOR expression, reduced anti-apoptotic proteins, increased apoptotic proteins including p53, cytochrome c, Caspase-3, and Bax, and enhanced granulosa-cell apoptosis (49). These findings are important for mechanism-based review writing because they demonstrate in actual DOR-derived granulosa cells that impairment of PI3K/Akt/mTOR signaling is not merely a theoretical aging-related change, but a pathway directly associated with defective granulosa-cell survival and diminished ovarian function (49).

In parallel, broader ovarian aging literature supports the pathogenic significance of this pathway. A recent review on ovarian aging emphasized that dysregulated PI3K/AKT/mTOR-related signaling contributes to premature follicle activation, granulosa-cell dysfunction, and accelerated reserve depletion, while FOXO-, sirtuin-, and AMPK-related crosstalk shapes the balance between survival, oxidative stress resistance, and reproductive longevity (7, 42). In addition, experimental ovarian injury studies show that oxidative stress can induce apoptosis and autophagy together with downregulation of PI3K, AKT, and mTOR, thereby contributing to ovarian dysfunction (18). These auxiliary data reinforce the interpretation that the PI3K/Akt/mTOR cascade is not only a survival pathway at the cellular level, but also a central signaling axis linking oxidative stress, follicular homeostasis, and ovarian aging (16, 18).

Further mechanistic support is provided by oxidative stress-driven granulosa-cell injury models. For example, miR-484 has been shown to contribute to DOR by impairing granulosa-cell function through YAP1-mediated mitochondrial dysfunction and apoptosis (52). In addition, miR-484 mediates oxidative stress-induced ovarian dysfunction and promotes granulosa-cell apoptosis via SESN2 downregulation (53). Although this work does not directly center on PI3K/Akt/mTOR, it underscores the broader biological principle that granulosa-cell mitochondrial injury and apoptosis are upstream determinants of ovarian dysfunction and reserve decline (17). Therefore, monomeric compounds capable of stabilizing mitochondria, reducing oxidative burden, and restoring survival pathways are biologically plausible therapeutic candidates for DOR (15, 17, 51, 54).

The PI3K/Akt/mTOR axis is bidirectional and cell-context dependent. In injured granulosa cells, appropriate PI3K/Akt activity can support survival, steroidogenesis, mitochondrial recovery, and anti-apoptotic signaling. In primordial follicles, however, sustained PI3K/Akt/mTOR activation can release FOXO3-mediated quiescence, promote premature follicle activation, and accelerate depletion of the finite reserve. Conversely, mTOR inhibition may preserve the primordial pool in overactivation models but could impede granulosa-cell survival or repair if excessive or applied during an acute injury phase. Apparently conflicting reports should therefore be interpreted through the experimental model, ovarian cell type, compound concentration or systemic dose, exposure duration, baseline pathway state, and intervention timing. The therapeutic objective is restoration of pathway homeostasis, not uniform activation or inhibition. Current evidence remains predominantly associative and requires cell-type-resolved perturbation, rescue experiments, and ovarian exposure measurements (7, 15, 16, 49, 51).

### 3.5 Ferroptosis and emerging forms of programed cell death

In recent years, ferroptosis has emerged as an important mechanism potentially contributing to ovarian aging and DOR. Unlike apoptosis, ferroptosis is a regulated form of cell death driven primarily by iron-dependent lipid peroxidation, glutathione depletion, and inactivation of glutathione peroxidase 4 (GPX4). Increasing evidence from human granulosa cells and animal models indicates that ferroptotic stress is closely associated with reduced ovarian reserve, impaired follicular development, and deterioration of oocyte quality. In granulosa cells from women with DOR and advanced age, GPX4 expression and intracellular glutathione levels are significantly decreased, whereas malondialdehyde (MDA) levels and abnormal mitochondrial morphology are increased, supporting a direct relationship between ferroptosis and ovarian functional decline (55). More broadly, current reviews of female infertility and ovarian disease indicate that iron overload, transferrin receptor-mediated iron uptake, glutathione/GPX4 dysfunction, and lipid peroxide accumulation form a mechanistic axis through which ferroptosis impairs ovarian endocrine function, follicle maturation, and reproductive competence (56, 57).

The pathogenic significance of ferroptosis in ovarian reserve depletion is further strengthened by mechanistic studies in primary ovarian insufficiency models. BNC1 deficiency has been shown to trigger oocyte ferroptosis through the NF2-YAP pathway, accompanied by increased TFRC and ACSL4 expression, iron overload, lipid peroxidation, and excessive follicular atresia. Importantly, pharmacological inhibition of ferroptosis partially rescues ovarian weight, follicular atresia, estrous cyclicity, and lipid metabolic abnormalities in Bnc1-mutant mice, indicating that ferroptosis is not merely an epiphenomenon but a causally relevant driver of ovarian insufficiency (58). These findings are highly relevant to DOR-oriented review writing because they connect iron metabolism, Hippo/YAP signaling, and follicular depletion within a unified disease framework (57, 58).

Several bioactive monomers and small natural molecules have shown anti-ferroptotic potential in ovarian-related systems. Pterostilbene, a stilbene derivative structurally related to resveratrol, effectively protects human ovarian granulosa cells from H2O2-induced oxidative injury and ferroptosis by restoring cell viability, reducing iron accumulation and oxidative stress, preserving mitochondrial membrane potential, and activating the Nrf2/HO-1 pathway (59). Because Nrf2-centered signaling is an established negative regulator of ferroptosis, these findings suggest that pterostilbene may preserve granulosa-cell survival by reinforcing endogenous antioxidant defense and suppressing lipid peroxide-driven cell death (57, 59). Similarly, spermidine has been shown to alleviate ovarian oxidative damage *in vivo* and *in vitro*, improve endocrine function and ovarian reserve, reduce granulosa-cell apoptosis, and suppress ferroptosis through coordinated activation of the Nrf2/HO-1/GPX4 and Akt/FHC/ACSL4 pathways (27). Notably, spermidine restored GPX4 and ferritin heavy chain expression while reducing ACSL4 accumulation, thereby linking antioxidant defense, iron handling, and lipid peroxidation control in the protection of follicular cells (27).

Berberine provides additional support for the concept that herbal monomers may modulate ferroptosis-associated ovarian injury. In a dihydrotestosterone (DHT)-induced human granulosa-cell injury model, berberine alleviated impaired proliferation, apoptosis, inflammatory activation, oxidative stress, Fe^2+^ accumulation, and MDA elevation, while improving ferroptosis-related injury through the circ_0097636/miR-186-5p/SIRT3 pathway (60). Although this study was performed in a PCOS-related granulosa-cell model rather than DOR per se, it is still mechanistically informative because it demonstrates that a classical Chinese herbal monomer can suppress iron-dependent oxidative damage and ferroptosis-like changes in ovarian granulosa cells, thereby improving cell survival and mitochondrial homeostasis (60).

Curcumin may also be placed within this anti-ferroptotic framework, although the uploaded direct evidence comes from a non-ovarian disease model. In hepatolenticular degeneration model systems, curcumin reduced metal overload, improved mitochondrial morphology, restored antioxidant status, increased Nrf2, HO-1, and GPX4 expression, and inhibited ferroptosis; these effects were reversed by erastin or Nrf2 inhibition (61). While this is not direct ovarian evidence, it provides supportive mechanistic rationale that curcumin, as a bioactive herbal monomer, is capable of stabilizing the Nrf2/HO-1/GPX4 axis and suppressing iron-dependent lipid peroxidation. Given the centrality of this pathway in ovarian ferroptosis, such findings strengthen the plausibility of curcumin-like compounds as candidates for ferroptosis-targeted ovarian protection (56, 57, 61).

Beyond ferroptosis itself, emerging evidence indicates substantial crosstalk between ferroptosis and other forms of regulated cell death. Ferroptosis has been linked to ferritinophagy, an autophagy-dependent process that promotes ferritin degradation, expands the labile iron pool, and facilitates reactive oxygen species generation and lipid peroxidation (62). In ovarian disease contexts, review evidence further suggests that ferroptosis intersects with inflammatory signaling, mitochondrial dysfunction, and other stress-responsive death programs, thereby amplifying granulosa-cell injury and follicular loss (57). This is particularly relevant for DOR, where oxidative stress, inflammation, mitochondrial damage, and programmed cell death rarely occur in isolation. Instead, ferroptosis likely participates in a broader cell-death network that may include pyroptosis, necroptosis, and autophagy-related degeneration, together accelerating ovarian aging and reserve depletion (56, 57, 62).

Collectively, available evidence suggests that ferroptosis is an important mechanistic component of ovarian aging and DOR progression. Reduced GPX4 activity, glutathione depletion, iron overload, ACSL4-related lipid remodeling, and mitochondrial structural damage appear to converge on granulosa-cell dysfunction, impaired oocyte competence, and follicular atresia (55–58). Natural monomers and related bioactive small molecules—including pterostilbene, spermidine, berberine, and potentially curcumin—may counteract these processes by restoring iron homeostasis, enhancing GPX4-centered antioxidant defense, suppressing lipid peroxidation, and improving mitochondrial quality control (27, 59–61). Therefore, targeting ferroptosis and its crosstalk with other regulated cell-death pathways represents a promising strategy for delaying follicular depletion and preserving ovarian function in DOR.

### 3.6 Regulation of autophagy and mitophagy

Autophagy and mitophagy are essential for ovarian quality control because they remove damaged proteins and dysfunctional mitochondria, thereby preserving granulosa-cell survival, oocyte competence, and follicular homeostasis. In DOR and ovarian aging, impaired mitochondrial turnover is commonly associated with reduced PINK1/Parkin-related signaling, p62 accumulation, oxidative stress, mitochondrial dysfunction, and accelerated follicular loss (22, 63, 64). However, ovarian injury is context-dependent: insufficient mitophagy may aggravate aging-related degeneration, whereas toxic or stress-induced overactivation of autophagy/mitophagy may also damage granulosa cells and follicles (65, 66). Thus, the key therapeutic goal is not simple activation or inhibition, but restoration of autophagy–mitophagy balance (67, 68).

Evidence from uploaded studies shows that several monomers regulate this pathway in different directions. Hyperoside protected against cyclophosphamide-induced ovarian injury by suppressing HIF-1α/BNIP3-mediated excessive autophagy, reducing follicular loss, restoring mitochondrial membrane potential, and improving fertility-related outcome (22). Nicotinamide mononucleotide improved age-related DOR by enhancing mitophagy in granulosa cells and supporting mitochondrial homeostasis (63). Spermidine rejuvenated aged oocytes by promoting mitophagy, lowering ROS, and improving fertilization and embryo-development potential (64). Salidroside similarly enhanced mitophagy and improved oocyte competence in reproductively old mice (69). Mechanistic studies further showed that Nur77 activation promoted PINK1/Parkin-mediated mitophagy and improved ovarian aging phenotypes, whereas loss of PINK1 impaired mitophagy and accelerated ovarian aging, supporting a causal role of defective mitochondrial turnover in ovarian decline (70, 71). By contrast, melatonin alleviated toxic ovarian injury partly through modulation of autophagy/mitophagy markers, while studies in PCOS and BPA-exposed granulosa cells indicated that excessive PINK1/Parkin signaling or AMPK/mTOR/ULK1-driven autophagy can itself be pathogenic, and that suppression of this overactivation may also be protective (65, 66, 72).

Dose- and context-dependent findings provide an important counterweight to predominantly positive reports. Spermidine was protective at 10–15 mg/kg in an oxidative-injury study (27), and 50 mg/kg produced greater reproductive-aging benefit than 100 mg/kg in aged mice (64). By contrast, an acute supraphysiological 150 mg/kg intraperitoneal dose increased ovarian H_2_O_2_ and malondialdehyde, reduced total antioxidant capacity, catalase, and superoxide dismutase, and induced granulosa-cell apoptosis (73). Autophagy findings are likewise directionally heterogeneous: hyperoside suppressed HIF-1α/BNIP3-associated excessive autophagy after cyclophosphamide (22), whereas nicotinamide mononucleotide, lower-dose spermidine, and salidroside restored insufficient mitophagy in aging-related models (63, 64, 69). These observations support a non-monotonic therapeutic window and reinforce that dose, injury mechanism, cell type, treatment sequence, and baseline autophagic flux must be reported rather than assuming that pathway activation or inhibition is intrinsically beneficial.

Taken together, the available literature indicates that monomer-based therapy may preserve ovarian reserve through dynamic regulation of autophagy and mitophagy. Some compounds inhibit maladaptive overactivation, whereas others restore insufficient mitochondrial clearance; both mechanisms converge on maintaining mitochondrial integrity, limiting oxidative damage, and protecting granulosa cells and oocytes.

### 3.7 Current limitations and future perspectives

Cellular and animal experiments answer different questions and should not be treated as interchangeable. KGN cells and primary granulosa cells permit controlled concentration–response testing, pathway perturbation, and rescue experiments, but they usually model an acute single stressor, lack endocrine cyclicity and follicular architecture, and omit theca, stromal, vascular, immune, and oocyte–granulosa interactions; nominal concentrations may also exceed achievable ovarian exposure. Animal models integrate estrous cyclicity, circulating hormones, follicle counts, oocyte competence, fertility, systemic metabolism, and toxicity, but species differences and the selected injury mechanism can dominate the phenotype. Improvement in an animal endpoint does not by itself demonstrate direct target engagement, and a cell concentration cannot be equated with an oral or injected dose. Concordance across cell and animal models strengthens inference; discordant results should be investigated through exposure, cell type, injury stage, and timing rather than averaged into a single positive conclusion.

Overall, the preclinical evidence-support profile is favorable: 9 compounds were rated Excellent, 11 Good, and 4 Fair within the review-specific framework (**Table 1**). The strongest studies combine DOR-relevant ovarian outcomes with cross-model consistency, cell-animal concordance, dose-response assessment, multi-omics confirmation, or genetic/pharmacological pathway perturbation. Several reports would nevertheless benefit from clearer descriptions of randomization, blinding, prospective sample-size determination, complete outcome reporting, and ovarian pharmacokinetics. Label-free strategies such as drug affinity-responsive target stability (DARTS) and the cellular thermal shift assay (CETSA), together with affinity pull-down, competition, and genetic-rescue approaches, can further substantiate protein-ligand engagement under near-native conditions (74–76). Future studies that integrate these approaches with existing endocrine, follicular, mitochondrial, oocyte, and fertility outcomes should further strengthen the translational value of the field.

Second, clinical translation remains a major bottleneck. Most evidence for monomer-based ovarian protection is still derived from *in vitro* and animal studies, whereas clinical evidence in ovarian insufficiency-related conditions remains limited. The available systematic review on complementary therapies for POI indicates that current human evidence is scarce and generally of low certainty, and that complementary interventions should not replace standard hormone therapy (77). Although POI and DOR are not identical entities, this finding still highlights the broader translational gap between promising experimental results and validated clinical benefit in ovarian functional decline. In parallel, several representative monomers, including curcumin, berberine, and icariin, are all affected by unfavorable pharmacokinetic properties such as poor solubility, low oral bioavailability, rapid metabolism, and strong formulation dependence, which may markedly compromise reproducibility and therapeutic applicability (78–80). Accordingly, future studies should place greater emphasis on formulation optimization, pharmacokinetic characterization, tissue distribution, long-term safety, and dose–response evaluation.

Third, while monomer-based research is advantageous for mechanistic dissection, it also inevitably loses the multi-component and potentially synergistic nature of traditional herbal medicine. Network pharmacology studies suggest that traditional medicines often act through multiple compounds, targets, and pathways simultaneously, providing a systems-level framework for understanding cooperative pharmacology (81). At the same time, natural-product research has also emphasized that complex mixtures may exhibit not only synergy, but also additivity or antagonism, and that biological activity cannot always be reduced to a single dominant constituent (82). Thus, future studies should not simply oppose “single monomer” and “compound formula,” but should instead investigate inter-monomer cooperation, active-component combinations, and the mechanistic relationship between purified monomers and clinically used prescriptions.

Finally, emerging technologies are expected to substantially deepen mechanistic understanding in this field. Single-cell transcriptomic analysis has already revealed marked cell-type-specific changes during ovarian aging, including altered antioxidative programs in oocytes and granulosa cells, highlighting the importance of resolving ovarian heterogeneity at high resolution (83). More recently, spatial transcriptomics combined with single-cell sequencing has further demonstrated that ovarian function depends on spatially organized interactions among oocytes, granulosa cells, theca cells, stromal cells, and immune populations, thereby providing a more precise framework for identifying the true target cell types and microenvironmental contexts responsive to monomer intervention (84). In addition, organoid systems of the female reproductive tract provide a human-relevant ex vivo platform that better preserves tissue architecture, functional properties, and drug-response characteristics than conventional two-dimensional culture models (85). Taken together, future DOR research should progressively advance from pathway-level description toward an integrated strategy that combines direct target identification, multi-omics profiling, spatially resolved cell biology, organoid-based validation, and translational verification.

A staged translational roadmap is recommended. First, efficacy should be prospectively powered and replicated in at least two etiologically distinct DOR-relevant models with age-appropriate controls, randomization, blinded assessment, and predefined primary outcomes. Second, causal target engagement should be established by CETSA, DARTS, affinity pull-down, CRISPR or knockdown, and rescue experiments in the relevant ovarian cell type. Third, dose–exposure–response relationships, active metabolites, ovarian tissue distribution, formulation dependence, reproductive toxicity, and offspring outcomes should be characterized. Fourth, findings should be tested in human primary ovarian cells, organoids, or ex vivo tissue before carefully designed early-phase clinical studies. Until such evidence exists, these compounds should be regarded as preclinical candidates and should not replace established DOR evaluation or standard clinical care.

## 4 Conclusion

Using a mechanism-first classification, the available preclinical literature provides substantial support for the ovarian-protective potential of selected bioactive natural products predominantly derived from Chinese herbal medicines. Across DOR-related experimental systems, these compounds regulate interconnected processes involving redox defense, inflammation, DNA-damage responses and senescence, apoptosis, ferroptosis, and autophagy-mitophagy homeostasis (86, 87). Nine compounds achieved Excellent and 11 achieved Good evidence support under the review-specific framework, reflecting relevant ovarian outcomes strengthened by cross-model consistency, cell-animal concordance, dose-response evidence, causal pathway perturbation, or integrated multi-level validation. The available findings also indicate that PI3K/Akt/mTOR and autophagy should be interpreted as homeostatic networks whose optimal regulation depends on cell type, model, dose, timing, and baseline pathway state. Although ovarian pharmacokinetics, independent replication, and direct target engagement remain important priorities for selected compounds, the collective evidence identifies a strong group of candidates for deeper mechanistic investigation and staged translational development in DOR.

## References

[1] Zhang X Zhang N Dong Z Sun H Diao Z Li Y . The role of Chinese herbal medicine in diminished ovarian reserve management. J Ovarian Res. (2025) 18:90. doi: 10.1186/s13048-025-01669-4 40307895 PMC12042416

[2] Penzias A Azziz R Bendikson K Falcone T Hansen K Hill M . Testing and interpreting measures of ovarian reserve: a committee opinion. Fertil Steril. (2020) 114:1151–7. doi: 10.1016/j.fertnstert.2020.09.134 33280722

[3] Choi R Park W Chun G Lee SG Lee EH . Investigation of the prevalence of diminished ovarian reserve in Korean women of reproductive age. J Clin Med. (2023) 12:5099. doi: 10.3390/jcm12155099 37568500 PMC10419389

[4] Ju W Zhao Y Yu Y Zhao S Xiang S Lian F . Mechanisms of mitochondrial dysfunction in ovarian aging and potential interventions. Front Endocrinol (Lausanne). (2024) 15:1361289. doi: 10.3389/fendo.2024.1361289 38694941 PMC11061492

[5] Shi YQ Zhu XT Zhang SN Ma YF Han YH Jiang Y . Premature ovarian insufficiency: a review on the role of oxidative stress and the application of antioxidants. Front Endocrinol (Lausanne). (2023) 14:1172481. doi: 10.3389/fendo.2023.1172481 37600717 PMC10436748

[6] Silva CA Yamakami LYS Aikawa NE Araujo DB Carvalho JF Bonfá E . Autoimmune primary ovarian insufficiency. Autoimmun Rev. (2014) 13:427–30. doi: 10.1016/j.autrev.2014.01.003 24418305

[7] Tesarik J Galán-Lázaro M Mendoza-Tesarik R . Ovarian aging: molecular mechanisms and medical management. Int J Mol Sci. (2021) 22:1371. doi: 10.3390/ijms22031371 33573050 PMC7866420

[8] Lliberos C Liew SH Zareie P La Gruta NL Mansell A Hutt K . Evaluation of inflammation and follicle depletion during ovarian ageing in mice. Sci Rep. (2021) 11:278. doi: 10.1038/s41598-020-79488-4 33432051 PMC7801638

[9] Cui X Li H Zhu X Huang X Xue T Wang S . CCDC134 enhances ovarian reserve function and angiogenesis by directly interacting with INHA in a mouse model of premature ovarian insufficiency. Apoptosis. (2025) 30:1311–30. doi: 10.1007/s10495-025-02092-2 40042746

[10] Chen Y Zhao Y Miao C Zhang Q Wang Z Li Z . Quercetin alleviates cyclophosphamide-induced premature ovarian insufficiency in mice by reducing mitochondrial oxidative stress and pyroptosis in granulosa cells. J Ovarian Res. (2022) 15:138. doi: 10.1186/s13048-022-01080-3 36572950 PMC9793602

[11] Barberino RS Lins T Monte APO Silva RLS Andrade KO Campinho DSP . Epigallocatechin-3-gallate attenuates cyclophosphamide-induced damage in mouse ovarian tissue via suppressing inflammation, apoptosis, and expression of phosphorylated Akt, FOXO3a and rpS6. Reprod Toxicol. (2022) 113:42–51. doi: 10.1016/j.reprotox.2022.08.010 35981663

[12] Xin L Li F Yu H Xiong Q Hou Q Meng Y . Honokiol alleviates radiation-induced premature ovarian failure via enhancing Nrf2. Am J Reprod Immunol. (2023) 90:e13769. doi: 10.1111/aji.13769 37766410

[13] Zhang M Yu X Li D Ma N Wei Z Ci X . Nrf2 signaling pathway mediates the protective effects of daphnetin against D-galactose-induced premature ovarian failure. Front Pharmacol. (2022) 13:810524. doi: 10.3389/fphar.2022.810524 35153783 PMC8832979

[14] Li X Li X Deng L . Chrysin reduces inflammation and oxidative stress and improves ovarian function in D-galactose-induced premature ovarian failure. Bioengineered. (2022) 13:8291–301. doi: 10.1080/21655979.2021.2005991 35311454 PMC9161991

[15] Xi H Wang Z Li M Duan X Li Y . Paeoniflorin promotes ovarian development in mice by activating mitophagy and preventing oxidative stress. Int J Mol Sci. (2024) 25:8355. doi: 10.3390/ijms25158355 39125927 PMC11313479

[16] Wu Q Chen M Li Y Zhao X Fan C Dai Y . Paeoniflorin alleviates cisplatin-induced diminished ovarian reserve by restoring the function of ovarian granulosa cells via activating FSHR/cAMP/PKA/CREB signaling pathway. Molecules. (2023) 28:8123. doi: 10.3390/molecules28248123 38138611 PMC10745843

[17] Chen C Li S Hu C Cao W Fu Q Li J . Protective effects of puerarin on premature ovarian failure via regulation of Wnt/β-catenin signaling pathway and oxidative stress. Reprod Sci. (2021) 28:982–90. doi: 10.1007/s43032-020-00325-0 32996063

[18] Yan Z Dai Y Fu H Zheng Y Bao D Yin Y . Curcumin exerts a protective effect against premature ovarian failure in mice. J Mol Endocrinol. (2018) 60:261–71. doi: 10.1530/jme-17-0214 29437881 PMC5863768

[19] He L Wang X Cheng D Xiong Z Liu X . Ginsenoside Rg1 improves pathological damages by activating the p21-p53-STK pathway in ovary and Bax-Bcl2 in the uterus in premature ovarian insufficiency mouse models. Mol Med Rep. (2021) 23:37. doi: 10.3892/mmr.2020.11675 33179093 PMC7684879

[20] Peng Y Sun L Guo W Liu Z Wang T Zou T . Berberine protects cyclophosphamide and busulfan-induced premature ovarian insufficiency in mouse model. J Pharmacol Sci. (2023) 153:46–54. doi: 10.1016/j.jphs.2023.07.004 37524454

[21] Li F Zhu F Wang S Hu H Zhang D He Z . Icariin alleviates cisplatin-induced premature ovarian failure by inhibiting ferroptosis through activation of the Nrf2/ARE pathway. Sci Rep. (2024) 14:17318. doi: 10.1038/s41598-024-67557-x 39068256 PMC11283570

[22] Zhu F Gao J Zeng F Lai Y Ruan X Deng G . Hyperoside protects against cyclophosphamide induced ovarian damage and reduced fertility by suppressing HIF-1α/BNIP3-mediated autophagy. BioMed Pharmacother. (2022) 156:113743. doi: 10.1016/j.biopha.2022.113743 36252358

[23] Tatone C Amicarelli F . The aging ovary—the poor granulosa cells. Fertil Steril. (2013) 99:12–7. doi: 10.1016/j.fertnstert.2012.11.029 23273984

[24] Melekoglu R Ciftci O Eraslan S Cetin A Basak N . Beneficial effects of curcumin and capsaicin on cyclophosphamide-induced premature ovarian failure in a rat model. J Ovarian Res. (2018) 11:33. doi: 10.1186/s13048-018-0409-9 29699594 PMC5918567

[25] Zhang Y Li X Liu R Huang X Yang Y Yuan J . Protective effect of bioactive components from Rubi fructus against oxidative damage in human ovarian granulosa cells induced by 2,2‐azobis (2‐methylpropionamidine) dihydrochloride. J Sci Food Agric. (2024) 104:4425–37. doi: 10.1002/jsfa.13330 38349056

[26] Haddad YH Said RS Kamel R Morsy EME El-Demerdash E . Phytoestrogen genistein hinders ovarian oxidative damage and apoptotic cell death-induced by ionizing radiation: co-operative role of ER-β, TGF-β, and FOXL-2. Sci Rep. (2020) 10:13551. doi: 10.1038/s41598-020-70309-2 32782329 PMC7419553

[27] Niu C Jiang D Guo Y Wang Z Sun Q Wang X . Spermidine suppresses oxidative stress and ferroptosis by Nrf2/HO-1/GPX4 and Akt/FHC/ACSL4 pathway to alleviate ovarian damage. Life Sci. (2023) 332:122109. doi: 10.1016/j.lfs.2023.122109 37741320

[28] Wang Y Pei H Chen W Du R Li J He Z . Deer blood hydrolysate protects against D-galactose-induced premature ovarian failure in mice by inhibiting oxidative stress and apoptosis. Nutrients. (2024) 16:3473. doi: 10.3390/nu16203473 39458468 PMC11510239

[29] Zeng Y Wang C Yang C Shan X Meng XQ Zhang M . Unveiling the role of chronic inflammation in ovarian aging: insights into mechanisms and clinical implications. Hum Reprod. (2024) 39:1599–607. doi: 10.1093/humrep/deae132 38906835

[30] Isola JVV Hense JD Osório CAP Biswas S Alberola-Ila J Ocañas SR . Reproductive ageing: inflammation, immune cells, and cellular senescence in the aging ovary. Reproduction. (2024) 168:e230499. doi: 10.1530/rep-23-0499 38744316 PMC11301429

[31] Wu C Chen D Stout MB Wu M Wang S . Hallmarks of ovarian aging. Trends Endocrinol Metab. (2025) 36:418–39. doi: 10.1016/j.tem.2025.01.005 40000274

[32] Castagnola L Gallino L Schafir A Vota D Grasso E Gori S . Ovarian premature aging: VIP as key regulator of fibro-inflammation and foamy macrophages generation. Mol Cell Endocrinol. (2025) 599:112486. doi: 10.1016/j.mce.2025.112486 39894337

[33] Navarro-Pando JM Alcocer-Gómez E Castejón-Vega B Navarro-Villarán E Condés-Hervás M Mundi-Roldan M . Inhibition of the NLRP3 inflammasome prevents ovarian aging. Sci Adv. (2021) 7:eabc7409. doi: 10.1126/sciadv.abc7409 33523841 PMC7775749

[34] Xu R Zhao H Qi J Yao G He Y Lu Y . Local glucose elevation activates pyroptosis via NLRP3 inflammasome in ovarian granulosa cells of overweight patients. FASEB J. (2023) 37:e22807. doi: 10.1096/fj.202201796RR 36826432

[35] Chen J Lin C Huang X Bian W . Baicalin enhances proliferation and reduces inflammatory-oxidative stress effect in H2O2-induced granulosa cells apoptosis via USP48 protein regulation. BMC Complement Med Ther. (2024) 24:42. doi: 10.1186/s12906-024-04346-z 38245760 PMC10799411

[36] Qu M Liu L Wang J Sui F Zhang M Wu X . Eriodictyol alleviates ovarian dysfunction in a mouse model of premature ovarian failure via the PI3K/Akt/NF-κB pathway and suppression of macrophage inflammation. Front Pharmacol. (2025) 16:1710805. doi: 10.3389/fphar.2025.1710805 41415564 PMC12708548

[37] Chen H Song L Xu X Han Z Peng F Zhang Q . The effect of icariin on autoimmune premature ovarian insufficiency via modulation of Nrf2/HO-1/Sirt1 pathway in mice. Reprod Biol. (2022) 22:100638. doi: 10.1016/j.repbio.2022.100638 35344846

[38] He S Li H Zhang Q Zhao W Li W Dai C . Berberine alleviates inflammation in polycystic ovary syndrome by inhibiting hyaluronan synthase 2 expression. Phytomedicine. (2024) 128:155456. doi: 10.1016/j.phymed.2024.155456 38537446

[39] Zhang X Cui X Ma F Zhou Y Song S Li Y . Molecular mechanisms and therapeutic potential of quercetin in ovarian functions. Phytomedicine. (2025) 144:156945. doi: 10.1016/j.phymed.2025.156945 40499220

[40] Xu X Wang Z Lv L Liu C Wang L Sun YN . Molecular regulation of DNA damage and repair in female infertility: a systematic review. Reprod Biol Endocrinol. (2024) 22:103. doi: 10.1186/s12958-024-01273-z 39143547 PMC11323701

[41] Teppa-Garrán A Pérez-Peña E Sobrevia L Marín R . Pharmacologic interventions targeting ovarian aging, cancer, and mitochondrial dysfunction: an updated evidence. Biochim Biophys Acta Mol Basis Dis. (2025) 1871:167987. doi: 10.1016/j.bbadis.2025.167987 40683607

[42] Wang X Wang L Xiang W . Mechanisms of ovarian aging in women: a review. J Ovarian Res. (2023) 16:67. doi: 10.1186/s13048-023-01151-z 37024976 PMC10080932

[43] Li N Wang J Wang X Sun J Li Z . Icariin exerts a protective effect against d-galactose induced premature ovarian failure via promoting DNA damage repair. BioMed Pharmacother. (2019) 118:109218. doi: 10.1016/j.biopha.2019.109218 31330441

[44] Jiang Y Zhang Z Cha L Li L Zhu D Fang Z . Resveratrol plays a protective role against premature ovarian failure and prompts female germline stem cell survival. Int J Mol Sci. (2019) 20:3605. doi: 10.3390/ijms20143605 31340581 PMC6678805

[45] Sevgin K Erguven P . SIRT1 overexpression by melatonin and resveratrol combined treatment attenuates premature ovarian failure through activation of SIRT1/FOXO3a/BCL2 pathway. Biochem Biophys Res Commun. (2024) 696:149506. doi: 10.1016/j.bbrc.2024.149506 38224665

[46] Ma LZ Wang A Lai YH Zhang J Zhang XF Chen SL . USP14 inhibition promotes DNA damage repair and represses ovarian granulosa cell senescence in premature ovarian insufficiency. J Transl Med. (2024) 22:834. doi: 10.1186/s12967-024-05636-3 39261935 PMC11389224

[47] Colella M Cuomo D Peluso T Falanga I Mallardo M De Felice M . Ovarian aging: role of pituitary-ovarian axis hormones and ncRNAs in regulating ovarian mitochondrial activity. Front Endocrinol (Lausanne). (2021) 12:791071. doi: 10.3389/fendo.2021.791071 34975760 PMC8716494

[48] Saccon TD Rovani MT Garcia DN Pradiee J Mondadori RG Cruz LAX . Growth hormone increases DNA damage in ovarian follicles and macrophage infiltration in the ovaries. Geroscience. (2022) 44:1071–81. doi: 10.1007/s11357-021-00380-8 33954912 PMC9135908

[49] Fan Y Tian D Lv Z Peng S Zhu S . LncRNA-THBS4 affects granulosa cell proliferation and apoptosis in diminished ovarian reserve by regulating PI3K/AKT/mTOR signaling pathway. J Reprod Immunol. (2025) 167:104419. doi: 10.1016/j.jri.2024.104419 39732055

[50] Zatalian N Dalman A Afsharian P Hezavehei M Gourabi H . Metformin protects prepubertal mice ovarian reserve against cyclophosphamide via regulation of the PI3K/Akt/mTOR signaling pathway and Yap-1. J Ovarian Res. (2024) 17:251. doi: 10.1186/s13048-024-01572-4 39702299 PMC11657643

[51] Cai J Li Y Zhao B Bao Z Li J Sun S . N-acetylcysteine alleviates D-galactose-induced injury of ovarian granulosa cells in female rabbits by regulating the PI3K/Akt/mTOR signaling pathway. Antioxidants (Basel). (2024) 13:384. doi: 10.3390/antiox13040384 38671832 PMC11047383

[52] Li H Wang X Mu H Mei Q Liu Y Min Z . Mir-484 contributes to diminished ovarian reserve by regulating granulosa cell function via YAP1-mediated mitochondrial function and apoptosis. Int J Biol Sci. (2022) 18:1008–21. doi: 10.7150/ijbs.68028 35173533 PMC8771835

[53] Wang X Yang J Li H Mu H Zeng L Cai S . miR-484 mediates oxidative stress-induced ovarian dysfunction and promotes granulosa cell apoptosis via SESN2 downregulation. Redox Biol. (2023) 62:102684. doi: 10.1016/j.redox.2023.102684 36963287 PMC10060268

[54] Jalouli M Mofti A Elnakady YA Nahdi S Feriani A Alrezaki A . Allethrin promotes apoptosis and autophagy associated with the oxidative stress-related PI3K/AKT/mTOR signaling pathway in developing rat ovaries. Int J Mol Sci. (2022) 23:6397. doi: 10.3390/ijms23126397 35742842 PMC9224321

[55] Hu J Wang H Fang J Jiang R Kong Y Zhang T . Ovarian aging‐associated downregulation of GPX4 expression regulates ovarian follicular development by affecting granulosa cell functions and oocyte quality. FASEB J. (2025) 39:e70469. doi: 10.1096/fj.202401580rr 40100097

[56] Zhang J Su T Fan Y Cheng C Xu L LiTian . Spotlight on iron overload and ferroptosis: Research progress in female infertility. Life Sci. (2024) 340:122370. doi: 10.1016/j.lfs.2023.122370 38141854

[57] Yao Y Wang B Jiang Y Guo H Li Y . The mechanisms crosstalk and therapeutic opportunities between ferroptosis and ovary diseases. Front Endocrinol (Lausanne). (2023) 14:1194089. doi: 10.3389/fendo.2023.1194089 37564979 PMC10411981

[58] Wang F Liu Y Ni F Jin J Wu Y Huang Y . BNC1 deficiency-triggered ferroptosis through the NF2-YAP pathway induces primary ovarian insufficiency. Nat Commun. (2022) 13:5871. doi: 10.1038/s41467-022-33323-8 36198708 PMC9534854

[59] Chen X Song QL Li ZH Ji R Wang JY Cao ML . Pterostilbene ameliorates oxidative damage and ferroptosis in human ovarian granulosa cells by regulating the Nrf2/HO-1 pathway. Arch Biochem Biophys. (2023) 738:109561. doi: 10.1016/j.abb.2023.109561 36898621

[60] Wang S Wang Y Qin Q Li J Chen Q Zhang Y . Berberine protects against dihydrotestosterone-induced human ovarian granulosa cell injury and ferroptosis by regulating the Circ_0097636/MiR-186-5p/SIRT3 pathway. Appl Biochem Biotechnol. (2024) 196:5265–82. doi: 10.1007/s12010-023-04825-y 38153651

[61] Sun X Zhang X Yan H Wu H Cao S Zhao W . Protective effect of curcumin on hepatolenticular degeneration through copper excretion and inhibition of ferroptosis. Phytomedicine. (2023) 113:154539. doi: 10.1016/j.phymed.2022.154539 36898256

[62] Stockwell BR Jiang X Gu W . Emerging mechanisms and disease relevance of ferroptosis. Trends Cell Biol. (2020) 30:478–90. doi: 10.1016/j.tcb.2020.02.009 32413317 PMC7230071

[63] Huang P Zhou Y Tang W Ren C Jiang A Wang X . Long-term treatment of nicotinamide mononucleotide improved age-related diminished ovary reserve through enhancing the mitophagy level of granulosa cells in mice. J Nutr Biochem. (2022) 101:108911. doi: 10.1016/j.jnutbio.2021.108911 34801690

[64] Zhang Y Bai J Cui Z Li Y Gao Q Miao Y . Polyamine metabolite spermidine rejuvenates oocyte quality by enhancing mitophagy during female reproductive aging. Nat Aging. (2023) 3:1372–86. doi: 10.1038/s43587-023-00498-8 37845508

[65] Zhou W Zhang J Lu X Zhao Z Weng Y Zhu C . Umbilical cord mesenchymal stem cell-derived extracellular vesicles improve excessive autophagy of granulosa cells through METTL3. Am J Physiol Cell Physiol. (2025) 328:C1586–604. doi: 10.1152/ajpcell.00785.2024 40106233

[66] Lin M Hua R Ma J Zhou Y Li P Xu X . Bisphenol A promotes autophagy in ovarian granulosa cells by inducing AMPK/mTOR/ULK1 signalling pathway. Environ Int. (2021) 147:106298. doi: 10.1016/j.envint.2020.106298 33387880

[67] Zhu X Li H Xue T Wang S Zhu R Luo J . Mechanistic study on the role of multi-pathway autophagy in ovarian aging: literature review. Apoptosis. (2025) 30:2694–721. doi: 10.1007/s10495-025-02181-2 40956551

[68] Samare-Najaf M Neisy A Samareh A Moghadam D Jamali N Zarei R . The constructive and destructive impact of autophagy on both genders’ reproducibility, a comprehensive review. Autophagy. (2023) 19:3033–61. doi: 10.1080/15548627.2023.2238577 37505071 PMC10621263

[69] Gu J Hua R Wu H Guo C Hai Z Xiao Y . Salidroside improves oocyte competence of reproductively old mice by enhancing mitophagy. Aging Cell. (2025) 24:e14475. doi: 10.1111/acel.14475 39789811 PMC12073897

[70] Yao Y Wang B Yu K Song J Wang L Zhang X . Nur77 improves ovarian function in reproductive aging mice by activating mitophagy and inhibiting apoptosis. Reprod Biol Endocrinol. (2024) 22:86. doi: 10.1186/s12958-024-01250-6 39044215 PMC11265396

[71] Zeng X Xie H Zhang W Yu Y Wang M Jiang Y . Loss of PINK1 impairs mitophagy and accelerates ovarian aging independent of Parkin. Free Radic Biol Med. (2026) 248:29–42. doi: 10.1016/j.freeradbiomed.2026.02.024 41707747

[72] Chouchene L Kessabi K Lajmi L Messaoudi I . Involvement of autophagy and mitophagy modulation in the protective effect of melatonin against BPS-induced ovarian toxicity. Reprod Toxicol. (2025) 138:109072. doi: 10.1016/j.reprotox.2025.109072 41005445

[73] Jiang D Jiang Y Long S Chen Z Li Y Mo G . Spermidine at supraphysiological doses induces oxidative stress and granulosa cell apoptosis in mouse ovaries. Theriogenology. (2021) 168:25–32. doi: 10.1016/j.theriogenology.2021.03.026 33845261

[74] Molina DM Jafari R Ignatushchenko M Seki T Larsson EA Dan C . Monitoring drug target engagement in cells and tissues using the cellular thermal shift assay. Science. (2013) 341:84–7. doi: 10.1126/science.1233606 23828940

[75] Chang J Kim Y Kwon HJ . Advances in identification and validation of protein targets of natural products without chemical modification. Nat Prod Rep. (2016) 33:719–30. doi: 10.1039/c5np00107b 26964663

[76] Lomenick B Jung G Wohlschlegel JA Huang J . Target identification using drug affinity responsive target stability (DARTS). Curr Protoc Chem Biol. (2011) 3:163–80. doi: 10.1002/9780470559277.ch110180 22229126 PMC3251962

[77] Maunder A Vermeulen N Vincent AJ Panay N Ee C . Complementary therapies for women with premature ovarian insufficiency: a systematic literature review to inform the 2024 update of the ESHRE/ASRM/IMS/CRE-WHiRL guidelines on premature ovarian insufficiency. Climacteric. (2026) 29:4–12. doi: 10.1080/13697137.2025.2530441 40719542

[78] Hegde M Girisa S BharathwajChetty B Vishwa R Kunnumakkara AB . Curcumin formulations for better bioavailability: What we learned from clinical trials thus far? ACS Omega. (2023) 8:10713–46. doi: 10.1021/acsomega.2c07326 37008131 PMC10061533

[79] Khoshandam A Imenshahidi M Hosseinzadeh H . Pharmacokinetic of berberine, the main constituent of Berberis vulgaris L.: A comprehensive review. Phytother Res. (2022) 36:4063–79. doi: 10.1002/ptr.7589 36221815

[80] Szabó R Rácz CP Dulf FV . Bioavailability improvement strategies for icariin and its derivates: A review. Int J Mol Sci. (2022) 23:7519. doi: 10.3390/ijms23147519 35886867 PMC9318307

[81] Yuan H Ma Q Cui H Liu G Zhao X Li W . How can synergism of traditional medicines benefit from network pharmacology? Molecules. (2017) 22:1135. doi: 10.3390/molecules22071135 28686181 PMC6152294

[82] Caesar LK Cech NB . Synergy and antagonism in natural product extracts: when 1 + 1 does not equal 2. Nat Prod Rep. (2019) 36:869–88. doi: 10.1039/c9np00011a 31187844 PMC6820002

[83] Wang S Zheng Y Li J Yu Y Zhang W Song M . Single-cell transcriptomic atlas of primate ovarian aging. Cell. (2020) 180:585–600.e19. doi: 10.1016/j.cell.2020.01.009 32004457

[84] Jones ASK Hannum DF Machlin JH Tan A Ma Q Ulrich ND . Cellular atlas of the human ovary using morphologically guided spatial transcriptomics and single-cell sequencing. Sci Adv. (2024) 10:eadm7506. doi: 10.1126/sciadv.adm7506 38578993 PMC10997207

[85] Alzamil L Nikolakopoulou K Turco MY . Organoid systems to study the human female reproductive tract and pregnancy. Cell Death Differ. (2021) 28:35–51. doi: 10.1038/s41418-020-0565-5 32494027 PMC7852529

[86] Ju W Zhao S Li D Zhang J Xiang S Lian F . Targeting programmed cell death with natural products: a potential therapeutic strategy for diminished ovarian reserve and fertility preservation. Front Pharmacol. (2025) 16:1546041. doi: 10.3389/fphar.2025.1546041 40510420 PMC12158948

[87] Hu H Zhang J Xin X Jin Y Zhu Y Zhang H . Efficacy of natural products on premature ovarian failure: a systematic review and meta-analysis of preclinical studies. J Ovarian Res. (2024) 17:46. doi: 10.1186/s13048-024-01369-5 38378652 PMC10877904

[88] Hooijmans CR Rovers MM de Vries RBM Leenaars M Ritskes-Hoitinga M Langendam MW . SYRCLE’s risk of bias tool for animal studies. BMC Med Res Methodol. (2014) 14:43. doi: 10.1186/1471-2288-14-43 PMC4230647 24667063

